# The Active for Life Year 5 (AFLY5) school-based cluster randomised controlled trial protocol: detailed statistical analysis plan

**DOI:** 10.1186/1745-6215-14-234

**Published:** 2013-07-24

**Authors:** Debbie A Lawlor, Tim J Peters, Laura D Howe, Sian M Noble, Ruth R Kipping, Russell Jago

**Affiliations:** 1 School of Social and Community Medicine, University of Bristol, Bristol, UK; 2 School of Clinical Sciences, University of Bristol, Bristol, UK; 3 School for Policy Studies, University of Bristol, Bristol, UK

## Abstract

**Background:**

The Active For Life Year 5 (AFLY5) randomised controlled trial protocol was published in this journal in 2011. It provided a summary analysis plan. This publication is an update of that protocol and provides a detailed analysis plan.

**Update:**

This update provides a detailed analysis plan of the effectiveness and cost-effectiveness of the AFLY5 intervention. The plan includes details of how variables will be quality control checked and the criteria used to define derived variables. Details of four key analyses are provided: (a) effectiveness analysis 1 (the effect of the AFLY5 intervention on primary and secondary outcomes at the end of the school year in which the intervention is delivered); (b) mediation analyses (secondary analyses examining the extent to which any effects of the intervention are mediated via self-efficacy, parental support and knowledge, through which the intervention is theoretically believed to act); (c) effectiveness analysis 2 (the effect of the AFLY5 intervention on primary and secondary outcomes 12 months after the end of the intervention) and (d) cost effectiveness analysis (the cost-effectiveness of the AFLY5 intervention). The details include how the intention to treat and per-protocol analyses were defined and planned sensitivity analyses for dealing with missing data. A set of dummy tables are provided in Additional file 1.

**Discussion:**

This detailed analysis plan was written prior to any analyst having access to any data and was approved by the AFLY5 Trial Steering Committee. Its publication will ensure that analyses are in accordance with an a priori plan related to the trial objectives and not driven by knowledge of the data.

**Trial registration:**

ISRCTN50133740

## Background

The aims and details of data collection for Active For Life Year 5 (AFLY5) are provided in the main trial protocol paper, which was published in 2011 [1]. This detailed analysis plan is an update to that published protocol. It was written between November-December 2012, with this final version completed in January 2013. This final version of the analysis plan was approved by the AFLY5 Trial Steering committee on 31 January 2013, prior to any of the researchers who will analyse the data having access to any of them.

Whilst the AFLY5 RCT began in May 2011, no data had been seen by any of the authors of this analysis plan at the time of writing. This final version has been submitted to the Trial Steering Committee Chair – Prof. J. Cade – who will note any subsequent changes to it.

Data, for initial analyses will be released to analysts D.A. Lawlor and L. Howe in the second week in February 2013; they will begin quality control checks and main analysis 1 (see below) then, with input from T.J. Peters and R. Kipping in interpreting results.

## Update

The purpose of this update is to provide a detailed analysis plan of the effectiveness and cost-effectiveness of the AFLY5 intervention. This includes details of how variables will be quality control checked and the criteria used to define derived variables. Details of four key analyses are also provided: (a) effectiveness analysis 1 (the effect of the AFLY5 intervention on primary and secondary outcomes at the end of the school year in which the intervention is delivered); (b) mediation analyses (secondary analyses examining the extent to which any effects of the intervention are mediated via self-efficacy, parental support and knowledge, through which the intervention is theoretically believed to act); (c) effectiveness analysis 2 (the effect of the AFLY5 intervention on primary and secondary outcomes 12 months after the end of the intervention) and (d) cost effectiveness analysis (the cost-effectiveness of the AFLY5 intervention). The details include how the intention to treat and per-protocol analyses were defined and planned sensitivity analyses for dealing with missing data. A set of dummy tables are provided in Additional file 1. We begin by tabulating the outcome and mediators and then go onto the detailed analysis plan.

By writing and publishing this analysis plan prior to any analysts having access to AFLY5 data we hope to ensure that analyses are driven by a priori defined methods related to the trial objectives and not influenced by knowledge of the data.

### Outcome and mediation measurements

Table 1 lists the primary and secondary outcomes and the hypothesised mediators, and describes the type of variable for each of these.

**Table 1:** AFLY5 outcomes and mediators

**Variable**	**Type**	**Units/categories**	**Comments**
**Primary outcomes**			
Accelerometer assessed mean time per day spent doing moderate/vigorous physical activity (MVPA)	Continuous	Minutes	
Accelerometer assessed mean time per day spent in sedentary activity	Continuous	Minutes	
Self-reported (validated questionnaire) consumption of servings of fruit and vegetables	Count	Servings	Will be treated as a continuous variable
**Secondary outcomes**			
Self-reported (validated questionnaire) mean time spent screen-viewing on a week day	Continuous	Minutes	
Self-reported (validated questionnaire) mean time spent screen-viewing on a Saturday	Continuous	Minutes	
Self-reported (validated questionnaire) consumption of servings of snacks	Count	Servings	Will be treated as a continuous variable
Self-reported (validated questionnaire) consumption of servings of high fat food	Count	Servings	Will be treated as a continuous variable
Self-reported (validated questionnaire) consumption of servings of high energy drinks	Count	Servings	Will be treated as a continuous variable
Body mass index (BMI)	Continuous	z(SD)-score	Age and gender standardised
Waist circumference (WC)	Continuous	z(SD)-score	Age and gender standardised
General overweight/obesity	Binary	No	Derived from BMI using IOTF thresholds
		Yes	
Central overweight/obesity	Binary	No	Derived from WC using IDF criteria
		Yes	
**Potential mediators to be explored in secondary analyses**			
Self-reported (validated questionnaire) physical activity self-efficacy	Score in whole numbers	Range 26-130	Will be treated as a continuous variable
Self-reported (validated questionnaire) fruit and veg consumption self-efficacy	Score in whole numbers	Range 21-105	Will be treated as continuous variable
Child-reported (validated questionnaire) perceived maternal logistic support for physical activity	Score in whole numbers	Range 3-12	Will be treated as continuous variable
Child-reported (validated questionnaire) perceived paternal logistic support for physical activity	Score in whole numbers	Range 3-12	Will be treated as continuous variable
Child-reported (validated questionnaire) perceived maternal modelling of physical activity	Score in whole numbers	Range 5-20	Will be treated as a continuous variable
Child-reported (validated questionnaire) perceived paternal modelling of physical activity	Score in whole numbers	Range 5-20	Will be treated as a continuous variable
Child-reported (validated questionnaire) perceived maternal limitation of sedentary behaviour*	Score in whole numbers	Range 4-16	Will be treated as a continuous variable
Child-reported (validated questionnaire) perceived paternal limitation of sedentary behaviour*	Score in whole numbers	Range 4-16	Will be treated as a continuous variable
Child-reported (validated questionnaire) perceived parental modelling for healthy eating fruit and vegetable consumption^$^	Score in whole numbers	Range 12-48	Will be treated as a continuous variable
Child’s knowledge test related to intervention	Score in whole numbers	Range 0-9	Will be treated as a continuous variable

*IOTF* International Obesity Task Force; IDF: International Diabetes Federation.

*For sedentary behaviour we are not aware of any validated questionnaire assessing parental modelling of healthy sedentary behaviour for use in children and so have only collected information regarding maternal and paternal limiting of sedentary behaviour.

^$^For fruit and vegetable consumption at the time of preparing all data collection tools, we were not aware of any validated questionnaires that provided relevant information for mothers and fathers separately or for logistical support of healthy fruit and vegetable consumption for use in children. The data we have therefore is for ‘parents’ and is the child’s report of their parental perceived modelling.

### Detailed analysis plan

The detailed analysis plan is described in the following sections:

1. Quality control checking (QC) and cleaning of data and derivation of new variables from collected (raw) data.

2. Effectiveness analysis 1: The effect of the AFLY5 intervention on primary and secondary outcomes at the end of the school year in which the intervention is delivered.

3. Mediation analyses: Secondary analyses examining the extent to which any effects of the intervention are mediated via self-efficacy, parental support and knowledge, through which the intervention is theoretically believed to act.

4. Effectiveness analysis 2: The effect of the AFLY5 intervention on primary and secondary outcomes 12 months after the end of the intervention.

5. Cost effectiveness analysis: The cost-effectiveness of the AFLY5 intervention.

### Quality checking, cleaning data and deriving variables

Identical cleaning/QC and variable derivation procedures will be used for data that were collected at baseline (completed May–October 2011), first follow-up (completed May–September 2012) and second follow-up (planned for February-July 2013).

### Accelerometer data

Accelerometer data will be analysed using appropriate software (e.g. Kinesoft).

In both studies of children and adults different methods are used for deriving accelerometer outcome variables in different studies [2-7]. Differences occur in:

•The epoch (time) length of recorded bouts of data

•What period of records of consecutive zero movement/counts are taken to indicate a participant has removed the accelerometer (these periods are removed from the calculation of hours wear per day)

•Number of hours per day that are considered to provide valid wake-time wear for derivation of outcomes

•Number of days that the accelerometer should be worn to provide valid total wear time for derivation of outcomes

•The thresholds of counts per minute of activity that are used to define MVPA and sedentary behaviour

Whilst the considerable research in this area highlights how different decisions for these issues result in different mean levels of outcomes [2-7], we could find no evidence that considered what effect (if any) these differences might have on the results in epidemiological association studies or in intervention studies, such as AFLY5. In a well-conducted randomised controlled trial, we would expect all participant characteristics, other than the intervention, to be the same by randomised group (other than differences that may occur due to chance). Thus, the particular accelerometer criteria that are used for all participants in a RCT should not influence the effect of the intervention on outcomes. That is we would expect differences in characteristics such as return of accelerometer, wear-time, number of periods of a given time of consistent zero levels of activity, etc., to be similar between children from schools randomised to control and those randomised to the intervention. We will test this assumption in AFLY5 (see Section 2.2 and dummy table in Additional file 1: Table S1).

Consequently, in AFLY5 we have selected all criteria related to wear time on the basis of face validity relevant to our study population and consistent with the instructions given to the children about wearing the accelerometer, as suggested in a recent review [3].

Our thresholds for defining time spent in MVPA and in sedentary behaviour are based on a literature review conducted at the time of writing this protocol. A recent review of calibration and validation studies in children for determining the thresholds that should be used to define MVPA noted the poor quality of many of these [6]. Calibration studies of between one to nine participants suggested thresholds for MVPA of between 1,770 to 3,581 counts per minute [6]. External validation studies included more participants than the original calibration studies, but were still of small sample size (*n* = 30–206), often of poor quality and used a variety of comparison methods [6]. Fewer studies are available of thresholds for defining sedentary behaviour in children. The most recent, and the largest (*n* = 206) and methodologically most sound external validation study to date [7], suggested that the thresholds recommended by Evenson [8] were most valid. We will therefore use the Evenson thresholds for MVPA (≥2,296 counts per minute) and sedentary behaviour (0–100 counts per minute) in AFLY5.

Further considerations in choosing which criteria to use for deriving accelerometer outcomes are the importance of minimising chance findings due to multiple testing and of potentially compromising statistical efficiency by excluding too many participants for not having valid wear data. We will compare accelerometer characteristics by randomised group to test our assumption that these are similar.

#### Criteria that will be used in AFLY5 for deriving accelerometer outcome measurements

•Data collected in 10-s epochs

•A period of ≥60 min of consecutive 0 counts assumed to be non-wear and these periods removed from the measurement of wear-time

•≥ 8 h per day to be considered to have valid wear-time for a given day

•≥ 3 valid days in total

•MVPA defined as ≥2,296 counts per minute

•Sedentary behaviour defined as 0 to 100 counts per minute

Once the key outcomes – time spent in MVPA and in sedentary behaviour – have been derived using the accelerometer software they will be exported from that software into Stata, and ‘general’ cleaning and checking of the distributions of these variables will be undertaken.

#### Cleaning/QC of the accelerometer derived variables will include

•Normal plots, histograms and scatter plots will be used to identify potentially implausible measurements.

•Scatter plots will compare for each variable its baseline and follow-up value and also will compare different variables measured at the same time point that would be expected to be moderately to strongly correlated; time spent in MVPA to time spent in sedentary behaviour, time spent in MVPA to weight and time spent in sedentary behaviour to weight.

•Values that appear outside of the main distribution in the majority of participants (i.e. outliers) on normal plots and histograms will be assumed to be correct if the scatter plots show consistency – e.g. lying close to the main ‘line’ of positive association for the same variable measured at baseline and again at follow-up or the inverse association of time in MVPA with time in sedentary behaviour and close to the main ‘line’ of inverse association of time spent in MVPA with weight.

•Outliers that deviate from the ‘line’ of the scatter plots by 2 SD or more on either axis will be considered implausible.

•For implausible values, the original data will be checked in the accelerometer software to make sure criteria have been applied correctly and any errors will be corrected.

•For remaining implausible values a variable that indicates ‘possible implausible value of *X*’ (where *X* is the name of the variable that has a possible implausible value) will be derived.

•In the main effectiveness analyses we will complete analyses with all participants (including where they have a possible implausible value) included and again with participants excluded for analyses with a given outcome if their value for that outcome has been marked as possibly implausible.

•If removal of participants with possible implausible values results in a change of a magnitude that for that outcome would affect the interpretation/conclusion for that outcome then both sets of results will be reported; otherwise only the results with all included irrespective of ‘implausible value’ status will be reported.

### Diet data

Questionnaire responses are entered into a Microsoft Access database.

#### Initial cleaning

Is undertaken to assign each food to a food or drink category (e.g. fruit and vegetables) based on the range of spellings (including incorrect spelling) that were identified in the pilot study and during cleaning of the baseline data for this trial. We use an automated system in Microsoft Access that was developed for this purpose during the feasibility/pilot study for AFLY5 [9]. Any remaining words that cannot be deciphered after going through this system are explored using discussion with parents and/or teachers of children of a similar age and Internet searches (e.g. for the identification of new brand names of sweets and other items that are unfamiliar to the coders) to help identify what the item might be. Where necessary a second independent individual looks at remaining words that cannot be deciphered. Words that remain impossible to decipher after all attempts are possible servings of food/drink that cannot be included further in any analyses.

#### Coding of diet data

After initial cleaning, codes are applied that indicated which (if any) outcome category – fruit and vegetables, snacks, high fat foods and high energy drinks – each food item belongs to. These codes are allocated following the validated scoring system developed by the investigators who developed the questionnaire [9]. Codes are allocated by one individual, with a random 5% coded independently by a second individual. Discrepancies of greater than 5% in this second coding would trigger re-coding of all data and a detailed check of why inconsistencies have occurred. Full details of how each food item is coded are provided in Additional file 2.

#### Final cleaning

Final cleaning will include:

•Exploring the distribution of the diet score (number of servings) for each of the four types of food used as an outcome in our study (fruit and vegetables, snacks, high fat foods and high energy drinks) using bar charts.

•Checking implausibly high values (for any of these scores it is possible for a child to eat no portions on a day, whereas very high values are more likely to be implausible).

•A priori we consider implausibly high values > 8 portions/day for any single outcome.

•Any possible implausible values will be checked by going back to the original questionnaire responses and coding for that questionnaire, with corrections made as appropriate.

•For remaining implausible values a variable that indicates ‘possible implausible value of *X*’ (where *X* is the name of the variable that has a possible implausible value) will be derived.

•In the main effectiveness analyses we will complete analyses with all participants (including where they have a possible implausible value) included and again with participants excluded for analyses with a given outcome if their value for that outcome has been marked as possibly implausible.

•If removal of participants with possible implausible values results in a change of a magnitude that for that outcome would affect the interpretation/conclusion for that outcome then both sets of results will be reported; otherwise only the results with all included irrespective of ‘implausible value’ status will be reported.

### Screen viewing data

Cleaning/QC of the screen viewing data will include:

•Normal plots, histograms and scatter plots will be used to identify potentially implausible measurements.

•Scatter plots will compare self-reported time spent screen-viewing at baseline to the same at follow-up and will also compare self-reported time spent screen-viewing at both time points on weekdays to that on Saturdays and also both to time spent in sedentary behaviour based on the accelerometer data.

•Values that appear outside of the main distribution in the majority of participants (i.e. outliers) on normal plots and histograms will be assumed to be correct if the scatter plots show consistency.

•Outliers that deviate from the ‘line’ of the scatter plots by 2 SD or more on either axis will be considered implausible.

•For implausible values, the original data will be checked on the completed questionnaires and any transcription errors corrected.

•For remaining implausible values a variable that indicates ‘possible implausible value of *X*’ (where *X* is the name of the variable that has a possible implausible value) will be derived.

•In the main effectiveness analyses we will complete analyses with all participants (including where they have a possible implausible value) included and again with participants excluded for analyses with a given outcome if their value for that outcome has been marked as possibly implausible.

•If removal of participants with possible implausible values results in a change of a magnitude that for self-reported screen viewing would affect the interpretation/conclusion for that outcome then both sets of results will be reported; otherwise only the results with all included irrespective of ‘implausible value’ status will be reported.

### Anthropometric data

#### Cleaning of data

The following will be undertaken for QC and cleaning data:

•Normal plots, histograms and scatter plots will be used to identify potentially implausible measurements.

•Scatter plots will compare each measure at baseline to the equivalent measure at follow-up and will also compare weight to height, weight to waist and height to waist at each time point. Values that appear outside of the main distribution in the majority of participants (i.e. outliers) on normal plots and histograms will be assumed to be correct if the scatter plots show consistency.

•Outliers that deviate from the ‘line’ of the scatter plots by 2 SD or more on either axis will be considered implausible.

•The original data collection sheets for these values will be checked and if these show that the data have been incorrectly entered in the database this will be corrected.

•For remaining implausible values a variable that indicates ‘possible implausible value of *X*’ (where *X* is the name of the variable that has a possible implausible value) will be derived.

•In the main effectiveness analyses we will complete analyses with all participants (including where they have a possible implausible value) included and again with participants excluded for analyses with a given outcome if their value for that outcome has been marked as possibly implausible.

•If removal of participants with possible implausible values results in a change of a magnitude that for that outcome would affect the interpretation/conclusion for that outcome then both sets of results will be reported; otherwise only the results with all included irrespective of ‘implausible value’ status will be reported.

#### Derivation of variables

The age range of the participants in this study at any one time point of data collection is narrow, because they are all from the same school year. However, over time children will age by on average 3 years. Because of the marked variability of body mass index (BMI) and waist circumference (WC) with age and gender we will derive internally standardised z-scores (also known as standard deviation scores) at each time point of data collection as follows:

$$z-\text{scoreBMI}=\left(\text{oBMIag}–\text{mBMIag}\right)÷\mathrm{sdBMIag}$$

$$z-\text{scoreWC}=\left(\text{oWCag}–\text{mWCag}\right)÷\text{sdWCag}$$

Where:

oBMI*ag* is the observed BMI for a participant of a given gender and age (within 6 month age categories)

mBMI*ag* is the mean BMI for participants of the same gender and same age (within 6 month age categories) as a given participant for whom the score is being derived

sdBMI*ag* is the standard deviation of the mean BMI for participants of the same gender and same age (within 6 month age categories) as a given participant for whom the score is being derived

oWC*ag* is the observed WC for a participant of a given gender and age (within 6 month age categories)

mWC*ag* is the mean WC for participants of the same gender and same age (within 6 month age categories) as a given participant for whom the score is being derived

sdWC*ag* is the standard deviation of the mean WC for participants of the same gender and same age (within 6 month age categories) as a given participant for whom the score is being derived

The binary anthropometric outcomes will be derived using:

•International Obesity Task Force (IOTF) age- (in 6 months) and gender-specific thresholds for overweight and obesity derived from BMI in children (general overweight/obesity) [10].

•For WC any participant above the 90th percentile for age- and gender-specific values derived from UK relevant centiles [11] will be defined as having central overweight/obesity, as suggested by the International Diabetes Federation (IDF) [12].

### Self-efficacy variables

Physical activity self-efficacy is assessed with a 26-item scale with each item having a five-point (1 to 5) score option (higher values indicating greater self-efficacy) [13]. Thus, the possible range of total scores is 26–130.

Fruit and vegetable self-efficacy is assessed with a 21-item scale with each item having a five-point (1 to 5) score option (higher values indicating greater self-efficacy) [14]. Thus, the possible range of total scores is 21–105.

#### Initial cleaning – dealing with missing data and deriving score

For these self-efficacy scores there are generally two types of missing data: (1) complete missing data (i.e. because the child was not present in school when data were collected; so far no child who was present has refused to complete the questionnaire) and (2) partial missing data where the child has completed some but not all items of the questionnaire. This section describes how we will deal with partial missing data only and hence derive a score for all children who have completed some of the questionnaire. Dealing with complete missing questionnaire data is addressed in the main analyses sections under ‘dealing with missing data’ (see below).

For both scores we will initially check for item missing data – i.e. the extent to which a child who has completed some of the questions for a given score has left some of the items blank.

Since the questionnaire is completed by the children with the fieldworkers present in the classroom and emphasis placed on completing every item we anticipate that there will be very few missing data.

Where data appear to be missing the original questionnaires will be checked to make sure this is not a data entry error. Any such errors will be corrected.

From the baseline assessment of these self-efficacy measurements we know that missing data are rare – 85% have complete data for all items, 10% missing just one item, 3% missing two items and 2% missing three or more items.

To deal with item missing data we will do the following:

•Any participant missing three or more items will be identified with a variable (derived variable indicating ‘high level of missing data for *X*’, where *X* is the specific self-efficacy measure affected)

•For all participants (irrespective of how many items are missing) a final score that takes account of missing data will be generated as follows:

$$\text{Self}\;\text{efficacy}\;\text{score}=\sum \mathrm{Io}+\left(\mathrm{Nm}\times \left(\sum \mathrm{Io}÷\mathrm{No}\right)\right)$$

Where

Io = all observed items

No = number of observed items

Nm = number of missing items

i.e. the score is the total sum of all observed scores plus the sum of missing scores with missing scores replaced with the mean of observed scores. So for example for a child who has completed 22 items out of the 26 for physical activity efficacy and has a sum of these 22 completed items of 78 the final score will be 78 + (4 × (78 ÷ 22)) = 81.5

•In the secondary analyses when we are exploring the role of these self-efficacy variables as mediators we will complete analyses with all participants [including where they have a ‘high’ level of missing (defined as above – missing three or more items) for the self-efficacy variable being considered] included and again with participants excluded for a given analysis if they have a high level of missing for the self-efficacy variable.

•If removal of participants with ‘high levels of item missing data’ for a given self-efficacy variable results in a change of a magnitude that would affect the interpretation/conclusion for that mediator (or for its effect on an outcome) then both sets of results will be reported; otherwise only the results with all included irrespective of ‘high levels of item missing data’ status will be reported.

#### Final cleaning

The following will be undertaken:

•Normal plots, histograms and scatter plots will be used to identify potentially implausible measurements.

•Scatter plots will compare self-efficacy variables at baseline and follow-up and will also compare the following within each time point; physical activity and fruit and vegetable self-efficacy (which we would expect to be positively associated), physical activity self-efficacy with accelerometer-assessed time spent in MVPA and fruit and vegetable self-efficacy with total portions of fruit and vegetables consumed.

•Values that appear outside of the main distribution in the majority of participants (i.e. outliers) on normal plots and histograms will be assumed to be correct if the scatter plots show consistency.

•Outliers that clearly deviate from the ‘line’ of scatter plots will be considered implausible.

•The original data collection sheets for these values will be checked and if these show that the data have been incorrectly entered in the database this will be corrected.

•For remaining implausible values a variable that suggests ‘possible implausible value’ will be derived.

•In the main effectiveness analyses we will complete analyses with all participants (including where they have a possible implausible value) included and again with participants excluded for a given analysis if they have an implausible indicator for a particular outcome.

•As with the high levels of item missing data analyses we will compare the effect of the intervention on each self-efficacy mediator variable with and without those with ‘possible implausible values’ removed. If removal changes the size of the effect by an amount that would change the interpretation/conclusion of the results analyses with and without these participants removed will be presented; otherwise only those with the participants included.

### Parental support variables

The parental support questionnaire for physical activity/sedentary behaviour [15,16] has items that provide information (and scores) for the child’s self reported perception of:

(1) Maternal logistic support/encouragement for physical activity (3 items of 4 options; range of possible scores 3–12)

(2) Paternal logistic support/encouragement for physical activity (3 items of 4 options; range of possible scores 3–12)

(3) Maternal modelling (i.e. showing good practice in front of the child) of physical activity (5 items of 4 options; range of possible scores 5–20)

(4) Paternal modelling of physical activity (5 items of 4 options; range of possible scores 5–20)

(5) Maternal restriction of sedentary behaviour (4 items of 4 options; range of possible scores 4–16)

(6) Paternal restriction of sedentary behaviour (4 items of 4 options; range of possible scores 4–16)

The parental support questionnaire for fruit and vegetable consumption has items that provide information for parental modelling of healthy behaviour for both parents combined [17]. The questionnaire consists of 12 items each of which has 4 options and hence this score has a range of possible values from 4 to 48. We were unable to identify a validated questionnaire for parental logistic support of fruit and vegetable consumption that was suitable for children to complete and the questionnaire that we have for parental modelling is with both parents combined.

#### Initial cleaning – dealing with missing data and deriving score

For these parental support scores there are generally two types of missing data as described above for the self-efficacy scores (note whilst these questions relate to parental support all questions were completed by the children in the classroom with no input from parents).

For all seven scores (6 related to physical activity and 1 to fruit and vegetable consumption) we will initially check for missing data – i.e. the extent to which a child has not completed all of the items for each score.

Since the questionnaire is completed by the children with the fieldworkers present in the classroom and emphasis placed on completing every item, we anticipate that there will be minimal missing data. For the physical activity variables that are collected separately for both parents we anticipate missing data will be greater for fathers than mothers, as some children may have limited contact with their fathers. The baseline data collection confirms this, with complete data on physical activity/sedentary behaviour support scores for mothers being provided by 90-92% of participants and for fathers for 82-84% of participants.

Because there are only a small number of items for each of the physical activity/sedentary behaviour parental support scores and hence the range of options is small, to deal with the small number of participants we anticipate will have some partial missing data we will:

•Generate a mean score for each child irrespective of the number of items completed (e.g. if a child has completed all 3 of the logistic modelling items their score will be the sum of each score divided by 3; if they have completed only 2 their score will be the sum of the 2 divided by 2)

•Generate a variable that indicates some missing data for any of the scores (i.e. whether the child has 1 or more items missing for any of the physical activity/sedentary parental support scores they will be indicated as having some missing).

•In the main mediator analyses all participants will be included for any given physical activity/sedentary behaviour parental support score irrespective of whether they had some missing data or not. The analyses will then be repeated with those who had some missing data excluded from analyses with that particular score.

•If removal of participants with ‘high levels of item missing data’ for a given parental support variable results in a change of a magnitude that would affect the interpretation/conclusion for that mediator (or for its effect on an outcome) then both sets of results will be reported; otherwise only the results with all included irrespective of ‘high levels of item missing data’ status will be reported.

•For the parental modelling of fruit and vegetable consumption the number of items and range of potential scores is relatively large and we will approach item missing data in this variable in the same way as that for the self-efficacy variables described above, i.e.:

•Any participant missing three or more items for the parental modelling of fruit and vegetable score will be identified with a variable (derived variable indicating ‘high’ level of missing data).

•For all participants (irrespective of how many items are missing) a final score that takes account of missing data will be generated as follows:

$$\begin{array}{l} \text{Parental support score}=\sum \mathrm{Io}\\ \;+\left(\mathrm{Nm}\times \left(\sum \mathrm{Io}÷\mathrm{No}\right)\right) \end{array}$$

•Wherei.e. the score is the total sum of all observed scores plus the sum of the mean of observed scores for any with a missing score. So a child who has completed 11 items out of the 13 items for parental modelling of health fruit and vegetable consumption where the sum of these 13 completed items is 28 will have a final score of 28 + (2 × (28 ÷ 13)) = 30.2

Io = all observed items

No = number of observed items

Nm = number of missing items

•In the main mediator analyses we will complete analyses with all participants (including where they have a ‘high’ level of missing for the parental modelling of fruit and vegetable variable) included and again with participants excluded if they have high levels of missing for this variable.

•If removal of participants with ‘high levels of item missing data’ for the parental modelling of fruit and vegetables variable results in a change of a magnitude that would affect the interpretation/conclusion for this mediator (or for its effect on an outcome) then both sets of results will be reported; otherwise only the results with all included irrespective of ‘high levels of item missing data’ status will be reported.

#### Final cleaning of all parental support scores

The following will be undertaken:

•Means, median, SD, IQR and full range,together with normal plots and histograms will be examined. As with the physical activity/sedentary behaviour scores any values outside the possible range must be an error in summing/generating the final score and will therefore be checked by looking at the Stata code used for doing this.

•To check for implausible values within the range expected relationships between variables will be checked by looking at scatter plots between each of the variables when measured at baseline and at follow-up and also between variables at the same time point as follows: physical activity/sedentary behaviour parental support and the fruit and vegetable support scores, using scatter plots. Where these suggest unlikely values for any participant (deviation from the scatter predicted line of association of more than 2 SD on either axes) the data entered values for each item will be compared against the original questionnaires and any entry errors corrected.

•For remaining unlikely values after these checks a variable that indicates ‘possible implausible value’ will be derived.

•In the main effectiveness analyses we will complete analyses with all participants (including where they have a possible implausible value) included and again with participants excluded for a given analysis if they have an implausible indicator for a particular outcome.

•As with the high levels of item missing data analyses we will compare the effect of the intervention on each mediator variable with and without those with ‘possible implausible values’ removed. If removal changes the size of the effect by an amount that would importantly influence the interpretation or conclusion of results both sets of results will be presented; otherwise only the ones with no exclusion.

### Child’s knowledge

At the two follow-up assessments we will collect data on the child’s knowledge in relation to what they have been taught in the intervention schools as part of the intervention. This measurement was not originally planned but as a study team we felt it was important to test whether specific knowledge is greater in children from the intervention schools than in those from the control schools after the intervention. We developed a questionnaire that reflects knowledge that the intervention lessons and homework aim to provide the children with (Additional file 3). This was developed by the study team with feedback from year 5 teachers and piloting (to test whether the questions were understood) amongst children aged 8–10 who were known to members of the study team. The questionnaire consists of nine multiple-choice questions. Children are instructed to tick one answer only from the three choices provided for each question. The range of scores possible is therefore from 0 to 9. We will deal with possible item-missing data for this knowledge test in exactly the same way as that described above for the parental support of physical activity/sedentary activity. QC checks will also be similar to those described above, although here we have no baseline measure with which to check likely outliers.

### Effectiveness and mediation analyses

The next sections describe the methods that will be used to analyse the effect of the intervention and whether this effect is mediated by measurements that indicate the pathways through which the intervention should, in theory, operate.

Three separate analyses will be undertaken and most likely presented in separate research publications:

1. Effectiveness analyses 1, which determines the effect of the ‘immediate’ effect of the intervention – i.e. the effect on outcomes assessed at the end of the school year during which the intervention has been delivered.

2. Mediation analyses. These are secondary analyses as they were not planned at the time of submission of the grant application and were not taken into account in the trial sample size calculation. The justification for undertaking these analyses is provided below. They will examine the extent to which any immediate effect of the intervention is mediated by measurements of the pathways through which the AFLY5 intervention is theorised to work.

3. Effectiveness analyses 2, which determined the ‘long-term’ effect of the intervention – i.e. the effect on outcomes assessed 12 months after the end of the school year in which the intervention was delivered.

The underlying analytical approach is similar for each of these analyses and its detailed description is provided in Section 2.2. and Sections 2.3 and 2.4; the focus is on specific aspects of the analyses that are unique to analyses 2 and 3 above. Dummy (empty) tables for these analyses are provided as examples in Additional file 1.

Although we initially planned that these analyses would be done with the analysts blind to which schools were intervention and which were control, this will not be done (analysts will know which schools are which). This is because the per-protocol analyses can only be done by knowing the number of lessons taught in each intervention school and therefore removes any possible ‘blinding’ of the analyst.

Effectiveness analyses 1: the immediate effect of the AFLY5 intervention on primary and secondary outcomes (i.e. effect at the end of the school year of the intervention)

The main effectiveness analysis paper will be written according to the “Consort 2010: extension to cluster randomised trials” statement [18].

Table 2 summarises the objectives, methods and planned timelines for this analysis.

**Table 2:** Summary of analysis 1 – effectiveness at 12 months

**Objective**	**Main methods**	Analysts and timing*		Journal submit*
Determine the effect of the AFLY5 intervention on primary outcomes	ITT analysis	DAL	Feb –March 2013	Aug 2013
	Multivariable multi-level linear regression (continuously measured outcomes), with adjustment for baseline variables	LH		
		RRK		
		TJP		
Determine the effect of the AFLY5 intervention on secondary outcomes	ITT analysis	DAL	Feb – March 2013	Aug 2013
	Multivariable multi-level linear regression (continuously measured outcomes) and multivariable multi-level logistic regression (for the two binary – general and central overweight/obese – outcomes), with adjustment for baseline variables	LH		
		RRK		
		TJP		
Complete secondary analyses to determine effect in those who completed the intervention as intended	Per-protocol analysis, excluding children from the intervention schools in which fewer than 11 lessons were taught. Multivariable multi-level linear or logistic regression (as above), with adjustment for baseline variables	DAL	Mar – Apr 2013	Aug 2013
		LH		
		TJP		
Sensitivity analyses to determine whether any effect of the intervention on primary outcomes based on accelerometer data vary by weekend or weekday	ITT analysis	DAL	Mar – Apr 2013	Aug 2013
	Multivariable multi-level linear regression (continuously measured outcomes), with adjustment for baseline variables	LH		
		TJP		
***Additional secondary analyses***				
Complete secondary analyses explore whether associations differ by gender and area deprivation	Stratified analyses (by gender and separately by school area deprivation)	DAL	Mar – Apr 2013	Not for journal but will be reported to funder (see below)
		LH		
	ITT analysis	TJP		
	Multivariable multi-level linear or logistic regression (as above), with adjustment for baseline variables.			
	Test of interaction between gender × intervention and deprivation × intervention			

*These are as planned at the time of writing this document.

*ITT* intention to treat. Results will be kept confidential within the analyst team (DAL: Debbie Lawlor; RRK: Ruth Kipping; LH: Laura Howe; TJP: Tim Peters) until all of the second follow-up outcome data have been collected – i.e. mid-July 2013.

This analysis is primarily concerned with the ‘immediate’ effectiveness of the intervention, with outcomes assessed ~12 months after the baseline assessment/randomisation (which corresponds to the immediate end of the intervention period).

#### Comparison of baseline characteristics and extent of missing follow-up data between intervention and control groups

We will compare relevant summary statistics of baseline characteristics between participants in schools who were allocated to be an intervention school and those allocated to be a control group in order to determine whether any potentially influential imbalance has occurred (by chance) between these two groups. These comparisons will also include accelerometer characteristics, including wear-time, time with consecutive zero levels of activity, etc., to test our assumption that the characteristics that are used in criteria for deriving the accelerometer variables do not vary by randomised group (see Section 1 above).

For all continuous and score variables we will check distributions using histograms and normal plots to examine how close to normality these are before deciding which summary statistics to present (see bullet points below).

The comparisons between the two groups will be made by summarising variables in each group (randomised versus control schools):

•Continuous variables that we anticipate will have approximately normal distributions (likely to include age, accelerometer time spent in MVPA, time spent in sedentary behaviour, BMI z-scores, WC z-scores) will be presented as means and standard deviations (SD).

•Continuous variables/scores that we anticipate will not have an approximate normal distribution (likely to include self-reported time spent screen viewing, self-efficacy scores for both physical activity and fruit and vegetables, parental modelling scores for fruit and vegetables) will be presented as medians and interquartile ranges (IQR).

•Binary/categorical variables (general overweight/obesity, central overweight/obesity, school involvement in other health promoting activities and school area deprivation) will be presented as number (N) and percentage (%).

We will not compare baseline characteristics between the two groups with a statistical test (*p*-value) as any low values simply represent a type-1 error under the assumption that we have adequately randomly allocated participants [19]. As described in the general study protocol paper our procedures for randomly allocating schools to control or intervention were adequate [1].

Additional file 1: Table S1 illustrates how the comparison of baseline characteristics will be presented.

#### Dealing with missing data

##### Missing baseline data

Any child from a randomised school with any baseline measure is a recruited study participant.

**Figure 1:**
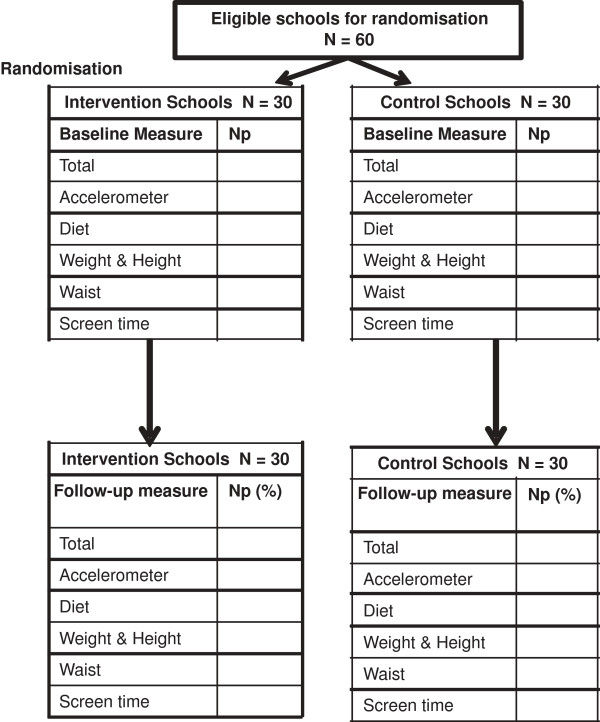
**Flow of schools and pupils through trial. ***Np* = Number of participants. For the outcome measurements the (%) is the% of participants for each measure who have an outcome measure out of those who had a baseline measure. Outcomes are grouped by collection type – e.g. all participants with valid accelerometer data will have time spent in MVPA and time spent in sedentary behaviour; those with weight and height will have BMI z-score and general overweight/obesity.

Numbers with valid data for each of the baseline measurements are likely to vary. For example, numbers with accelerometer data are likely to be lower than for other measurements because some participants will not have worn their accelerometer for sufficient time for data to be valid and some may not return their accelerometer. Numbers with BMI and WC measurements may be lower than for the dietary outcomes because some children may not provide assent for these measures. We anticipate that proportions with missing data for any particular measure will be similar in the two randomised groups but will check this (see Figure 1).

#### Intention-to-treat analyses and dealing with missing follow-up outcome data

For the main analyses we will use intention to treat (ITT). ITT requires all participants in a clinical trial to be included in the main analyses in the groups to which they were randomised [20,21]. This is straightforward if there is no loss to follow-up or missing data on some outcomes at follow-up amongst those who have been randomised, but is less straightforward where there is loss to follow-up/missing data [20,21]. A four-point framework for dealing with missing outcome data has recently been proposed to deal with this issue [20,21]. It emphasises the fact that all approaches (including complete case analysis – i.e. only including those with observed outcome data) – rely on assumptions that in any given situation may be more or less plausible but are always untestable. It therefore cautions against a ‘one size fits all’, but suggests using the most plausible assumptions about the nature of missing data and then testing these assumptions in sensitivity analyses; in particular it notes that in many cases the most plausible assumptions would support analyses on those with observed data using either mixed (multilevel models) or complete case analyses [20,21].

Complete case analyses and several of the common methods for imputing/dealing with missing data, including the multilevel linear regression model that we will use here for all primary outcomes, assume that missing data are missing at random (MAR). That is, missing values in participants with missing data are assumed to be similar to observed values in participants with similar levels of other variables that are observed in other participants (i.e. missing is independent of unobserved characteristics). Another way of thinking about this is that the effect of a randomised intervention is the same in those with missing data as in those without missing data. Having similar proportions of participants with missing data in each arm of a trial is reassuring with respect to the MAR assumption being correct, but is not a guarantee, as the plausible reasons for missing data in each arm could be different but result in similar proportions with missing.

In AFLY5, we minimise the extent of missing data through catch-up data collection – i.e. for each participating school at each phase of data collection there is a day for main data collection, but some children may be absent from school on that day; therefore for each school we have ‘catch-up’ days to obtain data on these children. As a result the likely reasons for missing follow-up data for ALL outcomes (i.e. where a child is not seen at all at one of the follow-up phases) are that the child moves school between data collection phases or the child is absent from school for a prolonged period or frequently so that they miss the main and catch-up data collection days. Missing one or more (but not all) of the specific measurements at follow-up could occur if the child does not give assent, or for the accelerometer-based outcomes the child does not return the accelerometer or does not wear it for a required period of time. In the case of the AFLY5 RCT, MAR is plausible since randomisation is at the level of the schools, parental opt-out consent is ascertained at the start of the study and relevant for all data collection times, and it is implausible that the delivery of the intervention lessons and homework in the intervention schools or lack of these in the control groups would affect the likelihood of a child being absent on days of data collection, declining assent for a particular measure or not returning the accelerometer or wearing to for the required time. Information from the local councils suggests that movement between schools is relatively low, but it is possible that children who move may differ from those who do not on the basis of unobserved characteristics. Children who move school might be from families who are relatively unorganised with children often moving school or they could be from families who move their child from state to private school in year 6 in order to attend private secondary school (Bristol has a higher than average for the UK proportion of children in private secondary school education). The possibility that these types of missing data might bias our findings if not explored will be assessed in sensitivity analyses (see sensitivity analyses 3 and 4 in Table 3).

**Table 3:** Dealing with missing data for main analyses and sensitivity analyses

	**Dealing with missing data**	**Assumptions**	**Implications/rationale**
**Main**^**a**^	All participants will be included if they have the particular outcome being assessed measured at the follow-up.	Data are MAR	The number included in these main analyses will differ for each outcome e.g. based on comments above regarding likely levels of missing data for each specific outcome measure it is possible that fewer participants will contribute to accelerometer outcomes than questionnaire outcomes
	An indicator variable (indicating whether baseline data are missing or not for each outcome) together with allocation of a ‘temporary’ value to those with baseline missing data, will be used to deal with missing baseline data [22]		
S1	Similar to above but participants are only included for each measurement if they have both baseline and follow-up data observed for each outcome	As above	Numbers will differ for each outcome.
			Allows assessment of whether those with missing baseline data differ in terms of the trial effect compared with those who do not have missing baseline data
S2	Similar to above but participants are only included if they have both baseline and follow-up data of all three primary outcomes	As above	For the three primary outcomes numbers will be the same numbers may differ for each secondary outcome.
			Allows assessment of whether any apparent differences in effect for the three primary outcomes are due to differs between these outcomes in missing data mechanisms
S3	Similar to the main analyses but for any child with a missing follow-up measure the child is allocated a value that is 10% ‘healthier’ for a given outcome than all participants with observed data (irrespective of randomised group). This will be done by calculating the 10% value of the mean or median follow-up measure for each outcome and then adding or subtracting (depending on whether healthier levels are higher or lower for the particular outcome) this value to the outcome mean or median; this final value will then be imputed to the outcome value for every child with missing follow-up data.	Those with missing outcome data on average behave in a relatively healthy way.	Numbers will be the same for all outcomes.
			Allows assessment of the possibility that missing data may be more likely to occur in families from higher SEP who may have missing data because of moving from state to private education. And to assess whether this form of missing data biases our assessment of the trial effect.
			This will also test whether selection bias occurs as a result of limiting analyses only to those with the required wear-time for the accelerometer based outcomes (this outcome is likely to have more missing data than other outcomes). As these analyses include all recruited participants.
S4	Similar to the main analyses but for any child with a missing follow-up measure the child is allocated a value that is 10% ‘less healthy’ for a given outcome than all participants with observed data (irrespective of randomised group). This will be done by calculating the 10% value of the mean or median follow-up measure for each outcome and then adding or subtracting (depending on whether less healthy levels are higher or lower for the particular outcome) this value to the outcome mean or median; this final value will then be imputed to the outcome value for every child with missing follow-up data	Those with missing data on average behave in less healthy ways than those who do not have missing data through mechanisms that are not captured by observed data	Numbers will be the same for all outcomes.
			Allows assessment of the possibility that missing data may be more likely to occur in families from lower SEP and who may have missing data because of being more dysfunctional and perhaps having to care for a relative at home or having higher rates of truancy. And to assess whether this form of missing data biases our assessment of the trial effect.
			This will also test whether selection bias occurs as a result of limiting analyses only to those with the required wear-time for the accelerometer-based outcomes (this outcome is likely to have more missing data than other outcomes). As these analyses include all recruited participants

^a^Note for other baseline characteristics that will be included in the model (gender, age and the school stratifying variables – school involvement in other health promoting activities and area deprivation) there should be no missing data. Thus, using a method that allows inclusion of those with missing baseline data in this analysis allows all recruited participants who have an outcome measure to be included in the analyses.

*S* Sensitivity analysis.

Table 3 details how missing baseline and follow-up outcome data will be managed in the main analyses and a series of sensitivity analyses that aim to test the assumptions regarding missing data.

In the main analyses we will use multilevel linear regression models accounting for the clustered nature of the data in AFLY5. For the main approach to all analyses any child with the measured outcome at follow-up will be included; we will do these analyses for each outcome separately so numbers included in the analyses between each outcome may vary. In order to include all children with the follow-up outcome measure (including those with a missing baseline value) and also be able to take account of the baseline value, we will use the method suggesting by White and Thompson for dealing with missing baseline values [22] in the first (immediate) effectiveness analyses and the mediation analyses. In the second (long-term) effectiveness analyses, we will use a repeated measures multilevel modelling approach to examine differences between randomised groups in the change in outcomes over time from baseline, and so missing data at baseline will be taken into account within these models. Both of these approaches assume data are MAR and in addition to these main analyses a number of sensitivity analyses will be undertaken; see Table 3.

#### Effect analyses

For the continuously measured outcomes (all primary outcomes and most of the secondary outcomes) we will:

•Use normal plots and histograms to assess normality of the follow-up measure of the outcome. If variables are approximately normally distributed they will be used as they are (i.e. with no transformation). If they are clearly non-normal we will explore transforming them to improve normality of the residuals in the regression models. The choice of whether or not to transform variables, and if so which transformation to use, will be decided by considering: (1) the distribution of the variable, (2) the distribution of residuals from regression models, (3) the ease of interpreting results following any given transformation compared with no transformation and (4) whether main results/conclusions are influenced by the transformation or not. From our pilot and feasibility studies for this trial and considerable experience with the outcome measurements that are used in this trial, we anticipate that all outcomes will be either approximately normally distributed or right (positively) skewed. For right skewed variables that result in markedly non-normal residuals in regression models we would use a natural log transformation and compare results with and without this transformation. If the overall conclusion is not altered by whether the variable is transformed or not, we would use the untransformed (easier to interpret) version. Where variables have been log-transformed, the resulting coefficients will be converted to differences in means on a % scale.

•Use multilevel multivariable linear regression to determine the difference in means between participants from schools allocated to the intervention and those allocated to control (reference group = control schools) whilst taking account of clustering (non-independence) amongst children from the same school.

•The analyses will include adjustment for the following baseline and stratifying covariables: age, gender, the baseline measure of the outcome being analysed (i.e. for the effect of the intervention on time spent in MVPA we will include baseline MVPA in the model and so on), school involvement in other health promoting activities and school area deprivation.

•The model for the main effect of the intervention on the continuously measured outcomes is

$$\begin{array}{l} Y_{\mathrm{ijp}}=\beta_{0}+\beta_{1}X_{1\mathrm{ijp}}+\beta_{2}X_{2\mathrm{ijp}}+\beta_{3}X_{3\mathrm{ijp}}+\beta_{4}X_{4\mathrm{ijp}}\\ \;+\beta_{5}X_{5\mathrm{ijp}}+\beta_{6}X_{6\mathrm{ijp}}+\beta_{7}X_{7\mathrm{ijp}}+\beta_{8}X_{7\mathrm{ijp}}\\ \;*X_{4\mathrm{ijp}}+C_{\mathrm{ij}}+{Є}_{\mathrm{ijp}} \end{array}$$

•Where

β_2_ to β_6_ are the adjusted associations of the baseline and stratifying covariables X_2*ijp*_ to X_6*ijp*_ with the outcome [i.e. age, gender, baseline measure of the outcome (X_4*ijp*_), school involvement in other health promoting activities and school area deprivation]

β_7_ is the association of the indicator variable X_7*ijp*_, indicating missing baseline measure of the outcome, with the follow-up outcome.

β_8_ is the interaction coefficient for the interaction of the missing baseline indicator variable with the baseline measure of the outcome (X_7ijp_*X_4*ijp*_)

C_*ij*_ is the school level effect for the school *j*th school in intervention group *i*

and C_*ij*_ ~ N(0, σ^2^_A_)

Є_*ijp*_ is the residual of the outcome for participant *p* from the *j*th school in intervention group *i*

and Є_*ijp*_ ~ N(0, σ^2^_W_)

and C_*ij*_ and Є_*ijp*_ are independent of each other.

Y_*ijp*_ is the outcome for participant *p* = 1…………………m, in the *j*th school *j* = 1 …………………..60 in intervention group *i* = 1, 2

β_0_ is the intercept, i.e. the outcome amongst those in intervention schools with the lowest level of all continuously measured covariables, the reference category for all categorical covariables and in school coded 1

β_1_ is the treatment effect (i.e. the mean difference in outcome comparing pupils from intervention schools to those form control schools) having adjusted for baseline characteristics and taken account of non-independence amongst children from the same school

$${Χ}_{1\mathrm{ijp}}=\left\{\begin{array}{l} 1\;\mathrm{if}\;i=1\left(\mathrm{intervention}\;\mathrm{school}\right)\\ 2\;\mathrm{if}\;i=0\left(\mathrm{control}\;\mathrm{school}\right) \end{array}\right)$$

For the binary outcomes (two secondary outcomes – general and central overweight/obesity):

•The approach will be broadly similar to that above described for continuously measured outcomes.

•A multilevel multivariable logistic regression model will be used to calculate the odds ratio of binary outcomes children in intervention schools to those in control schools (reference category), whilst taking account of clustering within schools.

•Baseline covariables identical to those listed above will be included.

•Thus, the model for binary outcomes is

$$\begin{array}{l} \pi_{\mathrm{ijp}}=\mathrm{Pr}\left(Y_{\mathrm{ijp}}=1\right)=\mathrm{logi}t^{-1}(\beta_{0}+\beta_{1}X_{1\mathrm{ijp}}\\ \;+\beta_{2}X_{2\mathrm{ijp}}+\beta_{3}X_{3\mathrm{ijp}}+\beta_{4}X_{4\mathrm{ijp}}+\beta_{5}X_{5\mathrm{ijp}}\\ \;+\beta_{6}X_{6\mathrm{ijp}}+\beta_{7}X_{7\mathrm{ijp}}+\beta_{8}X_{7\mathrm{ijp}}*X_{4\mathrm{ijp}}+C_{\mathrm{ij}}\\ \;+{Є}_{\mathrm{ijp}}) \end{array}$$

•Where

π_*ijp*_ is the probability that participant *p* = 1…………………m, in the *j*th school *j* = 1 …………………..60 in intervention group *i* = 1, 2 is overweight or obese

β_0_ is the intercept, i.e. the probability of normal weight amongst those in intervention schools with the lowest level of all continuously measured covariables, the reference category for all categorical covariables and in school coded 1

β_1_ is the treatment effect (i.e. the log odds of each binary outcome comparing pupils from intervention schools to those form control schools) having adjusted for baseline and stratifying covariables (as above) and taken account of non-independence amongst children from the same school

$${Χ}_{1\mathrm{ijp}}=\left\{\begin{array}{l} 1\;\mathrm{if}\;i=1\left(\text{intervention school}\right)\\ 2\;\mathrm{if}\;i=0\left(\mathrm{control school}\right) \end{array}\right)$$

β_2_ to β_6_ are the adjusted associations of the baseline and stratifying covariables X_2*ijp*_ to X_6*ijp*_ with the outcome (ie. age, gender, baseline measure of the outcome, school involvement in other health promoting activities and school area deprivation)

β_7_ is the association of the indicator variable X_7*ijp*_, indicating missing baseline measure of the outcome, with the follow-up outcome.

β_8_ is the interaction coefficient for the interaction of the missing baseline indicator variable with the baseline measure of the outcome (X_7ijp_*X_4*ijp*_)

C_*ij*_ is the school level effect for the school *j*th school in intervention group *i*

Є_*ijp*_ is the residual of the outcome for participant *p* from the *j*th school in intervention group *i*

For all nine secondary outcomes statistical significance will be indicated by a two-sided p-value of ≤ 0.01 (the equivalent of 0.05 following Bonferroni correction for multiple testing; the actual value of the Bonferroni correction 0.05/9 = 0.006, but we have rounded this up to 0.01) [1]. In order to aid interpretation we will present results by multiplying the p-value by 9 in journal publications for these secondary outcomes.

The trial sample size calculations for all outcomes took account of intraclass correlation coefficients calculated using our pilot/feasibility study data [1].

Empty example tables for the main and four sensitivity analyses of the effect of the intervention on primary and secondary outcomes are shown in Additional file 1: Tables S2 to S6.

#### Secondary per-protocol analyses to determine effect in those who completed the intervention as intended

A per-protocol analysis of the effect completing at least 70% of the lessons will be undertaken by:

•Including all children from control schools and only those children from intervention schools in which at least 70% of the lessons had been taught (i.e. at least 11 of the 16 lessons were taught).

•Children from schools that were randomised to the intervention but in which fewer than 11 lessons were taught will be excluded from these analyses.

•Teacher-completed logs will be used to determine how many of the lessons have been taught. Relevant data from these logs for completing the per-protocol analyses have been provided for 28 of the 30 schools. We will continue to try to obtain the other two, but it is possible we will not do so. In which case, we will undertake two secondary per-protocol analyses: one in which those who fail to return their logs are excluded (in effect treated as if they have taught fewer than 11 lessons) and one in which they are included (equivalent to assuming they have taught 11 or more of the lessons).

•Once children from schools that completed fewer than 70% of the lessons have been excluded the per-protocol analysis will be identical to the main analyses assessing the effectiveness of the intervention on the primary and secondary outcomes as described above, except that sensitivity analyses related to assumptions about missing data will not be completed, i.e. these secondary per-protocol analyses will only be conducted using the main analyses approach described above and in Table 3. Additional file 1: Table S7 illustrates how these results will be presented.

#### Sensitivity analyses to see if any effects on accelerometer assessed time spent in MVPA or sedentary behaviour vary by weekend or weekday

The main effect analyses, but not different sensitivity analyses for dealing with missing data, will be repeated for the accelerometer-assessed time spent in MVPA and sedentary behaviour outcomes separately for each outcome based on weekdays only and on weekend days only. For these analyses we will keep only those participants who have been included from the start of the effectiveness analyses on the basis of having worn the accelerometer for at least 3 days for at least 8 h. This means, for example, that a child who has just 3 days of adequate wear-time with all 3 being week days will contribute only to the week-day analysis (all 3 days contributing to those analyses), whereas if 2 days were weekdays and 1 a weekend day they would contribute to both weekday (with 2 days) and weekend (with 1 day) analyses. All aspects of these analyses (except for the way the outcomes are derived) will be the same as the main analyses described above.

#### Additional sensitivity analyses to explore whether there is any evidence that the intervention effect differs by gender and area deprivation

As noted in the grant application and study protocol paper [1], this RCT is not powered to be able to test the null hypotheses that the intervention works similarly in males and females and by different levels of area deprivation. However, the study funders (NIHR) are interested in whether there is any evidence that it might or not. Therefore the aim of these additional analyses is to explore whether there appears to be differences by gender or deprivation that are sufficiently large that they would warrant funding of a further RCT that was adequately powered to detect them or not. Such a situation would not only require a large difference but would really require the effect to work in one group at a level that would have public health importance but not work in another group (or even be detrimental in another groups). It is unlikely that if the intervention worked in both females and males but was somewhat stronger in one gender than another that would provide sufficient evidence to do a further RCT to confirm that or not as in practice it would be easier to provide the intervention to all children (irrespective of gender) and so such a trial would be unlikely to change practice. These analyses will be reported in the funder monologue (which is peer reviewed, publically available and widely disseminated), but we do not envisage including them in the main effect analysis journal publication due to the lack of statistical power and a concern that they would detract attention from the main results that the trial was powered to assess. These analyses will be done by:

•Repeating all of the main effectiveness analyses with primary and secondary outcomes as described above (main analysis only, see Table 3 above) separately in females and males, presenting the point estimates and their 95% CI in each subgroup.

•Undertaking an analysis that includes all participants (irrespective of gender) and includes an interaction term between gender and randomised group for each outcome. Presenting the interaction coefficient with its 95% confidence interval, as an indication of the precision with which this interaction can be detected in this trial and also presenting the p-value for the interaction effect.

•Repeating all of the main effectiveness analyses with primary and secondary outcomes as described above (main analysis only, see Table 3 above) separately in thirds (low, mid, high) of the school area deprivation score, presenting the point estimates and their 95%CI in each subgroup.

•Undertaking an analysis that includes all participants (irrespective of school area deprivation) and includes an interaction term between school area deprivation and randomised group for each outcome. Presenting the interaction coefficient with its 95% confidence interval, as an indication of the precision with which this interaction can be detected in this trial and also presenting the *p*-value for the interaction effect.

Additional file 1: Tables S8 and S9 illustrate how the results stratified by gender and school deprivation, respectively, will be presented.

### Mediation analyses: to examine the extent to which any immediate effect of the intervention is mediated by measurements of the pathways through which AFLY5 is theorised to work

These are secondary analyses as they were not planned at the time of submission of the grant application and were not taken into account in the trial sample size calculation. The justification of undertaking these analyses is that we feel that exploring whether the intervention has an effect on mediators that are relevant to this intervention is important for fully understanding the process by which the intervention may work or why it does not work if that turns out to be the case. For example we may find that the intervention is effective and that this is in part mediated by the child’s knowledge, but not by self-efficacy. Or we may find that the intervention does not work and also that it has no effect on any of the mediators, which would suggest either that it was poorly delivered or that it does not effectively work on the proximal characteristics that it is expected to work on. To balance the importance of looking at mediation with the fact that our original sample size calculation did not take account of this mediation analysis we consider these analyses to be exploratory and will take account of multiple testing for mediators in these analyses.

These analyses will be done after the immediate effectiveness analyses (described in Section 2.2 above) and will be published separately alongside qualitative analyses that will also explore whether the intervention worked in the way we would expect it to (see process evaluation plan). The mediation analyses will be conducted by Debbie Lawlor, Laura Howe and Tim Peters, with the expectation that a paper for publication will be submitted in November 2013.

Mediation will be assessed for the effect of the intervention on the primary outcomes only. This is because we have assessed child reported self-efficacy and parental support for these outcomes only. Mediation analysis assumes that the intervention influences the mediator(s) and through this influence on the mediator produces the effect on the outcome(s). Therefore the first stage in mediation analyses is to examine the effect of the intervention on the mediators (i.e. in these analyses mediators are treated as outcomes – dependent variables in the regression analyses).

To examine mediation we will

•First, determine the effect of the intervention on each of the ten measured mediators (see Table 1 above).

•Each of these mediators will be treated as a continuously measured variable and in the first stage we will explore the differences in mean scores of each mediator comparing children in the intervention to those in the control schools.

•Distributions of the mediators will be explored and procedures for transforming any that are non-normal will be the same as those used for the continuously measured primary and secondary outcomes, as described in Section 2.2 above.

•Multilevel multivariable linear regression will be used to examine the effect of the intervention on each mediator using the same approach as that used for the continuously measured primary and secondary outcomes, as described in Section 2.2 above.

•In these analyses we will include the following baseline and stratified covariables: age, gender, the baseline measure of each mediator, whether the school is involved in other health promoting activities and school level deprivation. Note: knowledge was not assessed at baseline so there is no baseline measure of this.

•Only the main approach for dealing with missing data (see Table 3 above) for any one of the mediators will be undertaken.

•As discussed in the section on cleaning these variables we will conduct analyses with and without those who have high levels (see above for definition) of item missingness within scales. Thus, for these analyses we will do one main analysis and one sensitivity analysis (with those who have high levels of item missing data for any mediator removed).

•In these analyses we will correct for multiple testing by adding the ten mediators to the nine secondary outcomes and assuming (two-sided) statistically significant effects with *p* ≤ 0.003 (0.05 ÷ 19). In journals we will present *p*-values multiplied by 19 to help interpretation.

•Second, we will explore whether mediators explain the effect of the intervention on outcomes. This second stage will only be conducted if: (1) the intervention has been shown to effect one or more of the primary outcomes (Section 2.2 above) and (2) the intervention has been shown to effect one or more of the mediators relevant to a primary outcome that the intervention has affected (first stage of mediation analyses described above).

•If the two criteria above are fulfilled we will complete multilevel multivariable linear regression exactly as described above in Section 2.2 for the specific outcome fulfilling these criteria. We will then repeat that analysis with any relevant mediator added and compare the effect of the intervention on the outcome before and after adjustment for the mediator.

•Each mediator that has been shown to be affected by the intervention, and that is relevant to an outcome that has also been affected by the intervention, will be added as a single covariable. In addition relevant mediators will then be added simultaneously in one final mediation model. For example, if the intervention is shown to increase time spent in MVPA, to increase knowledge relevant to the aims of the intervention and to increase child self-efficacy for physical activity (but has no impact on parental support for physical activity), we would complete the multilevel linear regression model with time spent in MVPA exactly as describe in Section 2.2 above. We would then repeat that analysis adding the following additional covariables: (1) knowledge score; (2) self-efficacy for physical activity score; (3) both knowledge and self-efficacy for physical activity score.

•A relative reduction (change towards the null) of the initial effect of the intervention on outcome (i.e. before addition of any mediators as covariables) of 10% or more will be considered to indicate some evidence of mediation.

•The% relative reduction in the initial trial effect on any of the primary outcomes will be recorded (see Additional file 1: Table S11) in all cases that fulfil the criteria for these analyses.

Additional file 1: Table S10 illustrates how the results of the analyses of the effect of the intervention on the mediators will be presented and empty table in Additional file 1: Table S11 shows how the results for the subsequent mediation analyses will be presented.

### Effectiveness analyses 2: the effect of the AFLY5 intervention on long-term outcomes (assessed ~12 months after the end of the intervention)

Table 4 summarises the objectives, methods and timelines for this second effectiveness analysis.

**Table 4:** Summary of analysis 2 – long-term effectiveness

**Objective**	**Main methods**	Analysts and timing*		**Journal submit**
Determine the effect of the AFLY5 intervention on primary outcomes assessed 12 months after the end of the intervention	ITT analysis	DAL LH TJP	Dec 2013 – Feb 2014	May 2014
	Multivariable multi-level linear regression (continuously measured outcomes), with adjustment for baseline variables.			
Determine the effect of the AFLY5 intervention on secondary outcomes assessed 12 months after the end of the intervention	ITT analysis	DAL	Dec 2013 – Feb 2014	May 2014
	Multivariable multi-level linear (continuously measured outcomes) or logistic (binary) regression, with adjustment for baseline variables.	LH		
		TJP		
Determine the effect of the AFLY5 intervention on change in primary outcomes between the baseline and the longer-term follow-up, including examining whether change in outcome between baseline and immediate follow-up differs from change in outcome between immediate and long-term follow-up.	ITT analysis	DAL	Dec 2013 – Feb 2014	May 2014
	Multivariable multi-level repeat measures linear regression, with adjustment for baseline variables.	LH		
		TJP		
Determine the effect of the AFLY5 intervention on change in secondary outcomes between the baseline and the longer-term follow-up, including examining whether change in outcome between baseline and immediate follow-up differs from change in outcome between immediate and long-term follow-up	ITT analysis	DAL	Dec 2013 –Feb 2014	May 2014
	Multivariable multi-level repeat measures linear regression (continuously measured outcomes) and multivariable multi-level logistic regression (binary outcomes), with adjustment for baseline variables	LH TJP		

* These are as planned at the time of writing this document.

This analysis is primarily concerned with the longer-term effectiveness of the intervention ~12 months after the end of the intervention period.

For these later follow-up analyses we will focus only on main associations with primary and secondary outcomes. We will not complete secondary per protocol and stratified (by gender and school based deprivation) analyses. This decision is made on the basis of the large number of analyses that will be undertaken for the first (immediately after intervention) effectiveness analyses and the importance of not ‘over analysing’ data. It is plausible that if the intervention is effective it will be more strongly effective in the short term. Therefore doing a very detailed analysis including secondary and stratified analyses at that time point is justified. The key aim of the analyses with the longer-term outcomes is to see if there is a sustained effect on the outcomes.

#### Analyses of difference in means and odds of outcomes at long-term follow-up between randomised groups

For the analyses where we examine the effect of the intervention at the long-term outcome (first two rows of the above table, the approach will be exactly the same as that described in Section 2.2 above, including completing the same main and sensitivity analyses and adjusting for the same set of baseline covariables. The tables will look similar to the empty tables in Additional file 1 that are referred to in Section 2.2 above.

#### Analyses of difference in change in outcomes between baseline and long-term follow-up between randomised groups

For the analyses of change in outcomes between baseline and the long-term follow-up we will use a multi-level model that fits repeat measurements (baseline, immediate follow-up, long-term follow-up) within each individual and allows a random effect (i.e. deviation from the study mean) of change for each individual. We will fit an interaction term with time so that we can explore whether differences between the two randomised groups in the change in outcome between baseline and immediate follow-up and then between immediate follow-up and long-term follow-up are consistent with each other.

In these models any participant with a measurement at any one of baseline, immediate or long-term follow-up can be included, and we will only do one set of analyses for this change measure with all participants included who have one of these time point assessments for a given outcome. Numbers will differ for each outcome.

Empty table in Additional file 1: Table S12 illustrates how the results of these long-term effectiveness analyses of change in outcomes between baseline and long-term follow-up will be presented.

### Economic evaluation analyses

This has been written by S. Noble.

The objective of the economic evaluation is to evaluate the difference in costs and the difference in effectiveness between the two arms of the trial. Only the costs relating to the delivery of the intervention and the intervention itself will be measured and valued, i.e. trial-related costs, e.g. data collection costs will not be included.

A cost consequence approach will be taken. This is when the differences in costs and consequences between the two arms of the trial are given in tabular form, and there is no attempt to estimate a summary score to encapsulate all the costs and benefits (e.g. the Incremental Cost effectiveness ratio). This approach is chosen given the number of important primary and secondary outcomes.

All costs will be valued in 2012/13 prices. As costs will be valued within the year no discounting of costs will take place.

These analyses will be undertake in late 2013/early 2014 with the timing of the cost-effectiveness publication to be with the publication of the paper reporting the long-term effectiveness outcomes.

### Primary analysis

The perspective will be that of the provider, schools and teachers.

The data for this primary analysis has mainly been collected through electronic time sheets, training day expense sheets and the teachers’ logs. The cost of each item of resource use will be evaluated as the resource use (e.g. number of hours) multiplied by its unit cost (see Table 5 for how resource use will be measured and valued). In the primary analysis it is assumed that no costs incur in the comparison arm. Costs per child will be estimated using the school specific information and allocating global costs equally amongst the children in the intervention schools.

**Table 5:** How resource use will be measured and valued for the primary analysis

**Resource**	**How it will be measured**	**How it will be valued**
***Global organisation of training***		
CH staff time organising training, including organising training materials and briefing the trainers^1^	TS: number of hours	Salary scales
CH staff attendance at training day^1^	TS: number of hours	Salary scales
Trainers fee^1^	Fee per session	Fee as given
Venue cost^1^	Cost per hour	University finance
Trainers subsistence cost^1^	From expense sheets	Cost as given
Refreshments^1^	From invoices	Cost as given
***School-specific organisation of training***		
CH staff time organising training^2^	TS: number of hours	Salary scales
CH staff time on phone calls^2^	TS: number of phone calls* average length of phone call (in min)	Salary scales
School staff time on phone calls^2^	TS: number of phone calls* average length of phone call (in min)	Salary scales
Phone calls^2^	TS: number of phone calls	BT
Teachers time attending training day^2^	Cost of supply teachers	Cost given by schools
Travel costs^2^	TDES: Car: mileage	University reimbursement
	Bus/train/taxi: fare	
Child care costs^2^	TDES	Cost given by teachers
Informal costs:	TDES: difference between normal travel time to work and travel to training day	Average wage rate from labour force survey
Extra time spent travelling to training day^2^		
***Global delivery of intervention***		
Time spent producing teaching and homework materials^1^	TS: number of hours	Salary scales
Cost of consumables^1^	TS	Cost as given
CH staff time in meetings in relation to delivering the intervention^1^	TS: number of hours	Salary scales
***School-specific delivery of intervention***		
Time spent delivering materials to schools^2^	TS: number of hours	Salary scales
Travel costs of delivering materials to schools^2^	Travel claim forms	University reimbursement
CH staff time corresponding with schools in relation to delivery of the intervention^2^	TS: number of hours	Salary scales
Phone calls^2^	TS: number of phone calls	BT
School staff time on phone calls^2^	TS: number of phone calls* average length of phone call (in min)	Salary scales
Teachers’ time in preparation of AFL5 lessons^2^	TL: number of minutes	Salary scales
The opportunity cost of teaching the AFL5 lessons^2^	TL: the AFL5 lesson time in min. Who taught the AFL5 lesson? The lesson it displaced. Who would have taught the displaced lesson?	The AFL5 lesson time (min)*pro rata salary scale of teacher delivering session minus the AFL5 lesson time (min)*pro rata salary scale of teacher who would have taught displaced lesson
Consumables used ^2^	TL	Cost as given

TDES (training day expense sheet: school staff); TS (time sheet); TL (teacher’s log).

^1^Divided by number of study pupils in AFL5 intervention arm.

^2^Divided by number of study pupils in each AFL5 intervention arm school.

The total cost of the intervention will be estimated by summing the cost of each item of resource use given in Table 5. An average cost per pupil for each school will be estimated in addition to an overall average cost per pupil.

### Secondary analysis

Parental questionnaires are being administered to the parents of both intervention and control groups for them to complete based on their experiences during the time of the intervention. What resources were collected and how they will be measured and valued are given in Table 6. As in the effectiveness analyses described above, multilevel multivariable linear regression will be used to determine the mean difference between participants in schools allocated to the intervention and those allocated to control (reference group = control schools) for the three economic variables listed below, whilst taking account of clustering (non-independence) amongst children from the same school. The main analyses will include adjustment for the stratification variables (level of school involvement in health promoting activities and school area deprivation) and child gender and baseline age.

**Table 6:** How will resource use be measured and valued for the secondary analysis

**Resources used**	**How will it be measured**	**How will it be valued**
Parental time spent on relevant homework	Parent questionnaire (min)	Average wage rate from labour force survey
Household spend on food	Parent questionnaire: cost per week	Cost given, adjusted for household members
Cost of out of school activities	Cost per week	Cost given, termly costs will be converted to weekly costs
Parental time spent on child activities	Parental questionnaire: (hours per week)	Average wage rate from labour force survey
NHS resource use for exercise related injuries	Number of visits/nights in hospital (parental questionnaire)	National reference costs
Private health service resource use for exercise-related injuries	Number of visits	Using available web based sources
Paid time off work because of exercise related injuries	Number of days	Average wage rate from labour force survey
Unpaid time off work because of exercise related injuries	Number of days	Average wage rate from labour force survey

Three different analyses will be conducted to estimate:

(1) Difference in cost of parental time

(2) Difference in consumable costs (out of school activities, food)

(3) Difference in NHS costs

It is likely that only a small proportion of the parental questionnaires will be returned.

The reasons for missingness will be explored, e.g. some parents may not have received the questionnaires. A complete case analysis will be initially conducted and then, depending on the type of missingness, other missing data techniques such as multiple imputation will be used. Any methodological or parameter uncertainty will be examined through a series of one-way sensitivity analyses for both the primary and the secondary analyses.

## Competing interests

The authors declare that they have no competing interests.

## Authors’ contributions

DAL developed the main content of the statistical analysis plan with input from TJP and LD. SMN wrote the economic evaluation analysis plan. RRK and RJ made key input in relation to definitions, cleaning and data management of the outcomes and mediators. DAL wrote the first draft of the plan, with the exception of the economic evaluation plan, which was written by SN. All authors commented on the draft. All authors read and approved the final manuscript.

## Supplementary Material

Additional file 1 Empty (dummy) tables illustrating how results will be presented. Click here for file

Additional file 2 **AFLY5 Diet Data Coding in Section A of Questionnaire.** Food and drink items for diet categories. Click here for file

Additional file 3 Knowledge assessment devised by study team. Click here for file
