# Supplementary material for: The Active for Life Year 5 (AFLY5) school-based cluster randomised controlled trial protocol: detailed statistical analysis plan
Source: Trials. 2013 Jul 24;14:234. doi: 10.1186/1745-6215-14-234 (PMC3733690; doi:10.1186/1745-6215-14-234)
Supplement: Additional file 1 — Empty (dummy) tables illustrating how results will be presented. [file 1745-6215-14-234-S1.doc]

**Additional file 1: Empty (dummy) tables illustrating how results will be presented**

Illustrative empty tables are presented for effectiveness analyses 1 (i.e. with outcomes assessed immediately after the intervention). All of the tables that are likely to be produced for this analysis are presented though many of them look very similar whilst they are empty. This allows the number of analyses that are planned to be appreciated. It also supports the key rationale of an a priori analysis plan by clearly demonstrating the set of results that a reviewer / reader would expect to see for these analyses. In the journal publication it is highly likely that several of these tables would be in on-line (web-only) supplementary material. But here they are presented in order that they are likely to be completed / are discussed in the main analysis plan document. Which appear in on-line supplementary material will depend on the final list of authors in negotiation with journal editors.

**Empty Table S1: Comparison of baseline characteristics by randomised group**

| **Characteristic** | **Unit and type of summary measure*** | **Intervention schools** | | **Control schools** | |
| --- | --- | --- | --- | --- | --- |
|  |  | Number | Distribution | Number | Distribution |
| Age | Mean (SD) months |  |  |  |  |
| MVPA | Mean (SD) minutes |  |  |  |  |
| Sedentary behaviour | Mean (SD) minutes |  |  |  |  |
| Servings of fruit and vegetables | Median (IQR) number / day |  |  |  |  |
| Servings of snacks | Median (IQR) number / day |  |  |  |  |
| Servings of high fat foods | Median (IQR) number / day |  |  |  |  |
| Servings of high energy drinks | Median (IQR) number / day |  |  |  |  |
| BMI | Mean (SD) z-score |  |  |  |  |
| WC | Mean (SD) z-score |  |  |  |  |
| Screen-viewing weekday | Median (IQR) minutes |  |  |  |  |
| Screen-viewing Saturday | Median (IQR) minutes |  |  |  |  |
| Total number of days of wearing accelerometer | Median (IQR) days |  |  |  |  |
| Number of weekdays of wearing accelerometer | Median (IQR) days |  |  |  |  |
| Total hours of wearing accelerometer | Mean (SD) hours / day |  |  |  |  |
| Hours of wearing accelerometer on weekdays | Mean (SD) hours / day |  |  |  |  |
| Periods of 60 minutes or more of consecutive zero activity | Median (IQR) number per day |  |  |  |  |
| **Categorical variables** | | | | | |
| Gender | N (%) female |  |  |  |  |
|  | N (%) male |  |  |  |  |
| General overweigh / obesity | N (%) No |  |  |  |  |
|  | N (%) Yes |  |  |  |  |
| Central overweigh/obesity | N (%) No |  |  |  |  |
|  | N (%) Yes |  |  |  |  |
| Return accelerometer | N (%) Yes |  |  |  |  |
|  | N (%) No |  |  |  |  |
| Wore accelerometer for requested amount of time | N (%) Yes |  |  |  |  |
|  | N (%) No |  |  |  |  |
| Wore accelerometer for required amount of time | N (%) Yes |  |  |  |  |
|  | N (%) No |  |  |  |  |
| School involved in other health promoting activities | N (%) Yes |  |  |  |  |
|  | N (%) No |  |  |  |  |
| School deprivation score | N (%) low |  |  |  |  |
|  | N (%) medium |  |  |  |  |
|  | N (%) high |  |  |  |  |

* Whether we present data as means (SD) or median (IQR) is based on our prior knowledge of the likely distribution of these variables, but this will be checked in these data. If variables are approximately normally distributed they will be presented as mean (SD); if not as median (IQR).

**Table S2: Main intention to treat analyses of the effect of AFLY5 intervention on primary and secondary outcomes assessed immediately after the end of the intervention**. Numbers vary by outcome as indicated in the table.

| **Outcome** | **Control group (reference group)** | | **Intervention group** | | **Main effect (group difference)** | | |
| --- | --- | --- | --- | --- | --- | --- | --- |
|  | **Number** | **Mean (SD), median (IQR) or number (%)** | **Number** | **Mean (SD), median (IQR) or number (%)** | **Number** | **Difference in means or odds ratio (95%C)** | **p-value** |
| **Continuously measured outcomes** | | | | | | | |
| **Time spent in MVPA (minutes per day)** |  |  |  |  |  |  |  |
| **Time spent in SB (minutes per day)** |  |  |  |  |  |  |  |
| **Servings of fruit and vegetables (number per day)** |  |  |  |  |  |  |  |
| Time spent screen-viewing (minutes per day weekday) |  |  |  |  |  |  |  |
| Time spent screen-viewing (minutes per day Saturday) |  |  |  |  |  |  |  |
| BMI (z(sd)-score) |  |  |  |  |  |  |  |
| WC (z(sd)-score) |  |  |  |  |  |  |  |
| Servings of snacks (number per day) |  |  |  |  |  |  |  |
| Servings of high fat foods (number per day) |  |  |  |  |  |  |  |
| Servings of high energy drinks (number per day) |  |  |  |  |  |  |  |
| **Binary measured outcomes** | | | | | | | |
| Generally overweight/obese |  |  |  |  |  |  |  |
| Centrally overweight/obese |  |  |  |  |  |  |  |

Outcomes in bold are primary outcomes (p ≤ 0.05 indicates statistical significance); all others are secondary outcomes (p ≤ 0.05 after multiplying the actual p-value by 9 (to adjust for multiple testing of secondary outcomes) indicates statistical significance)

All differences in means / odds ratios with their 95%CI have been estimated using a multi-level model to account for clustering (non-independence) among children from the same school. Multi-level multivariable linear regression was used for effects of the intervention on continuously measured outcomes and multi-level multivariable logistic regression was used for binary outcomes.

The following baseline / school stratifying covariables were included: age, gender, the baseline measure of the outcome under consideration, school involvement in other health promoting behaviours, school area level deprivation.

MVPA: moderate and vigorous physical activity (accelerometer assessed), SB: sedentary behaviour (accelerometer assessed), BMI: body mass index, WC: waist circumference, F&V fruit and vegetables.

In these analyses participants were included for each outcome if they had a follow-up measurement of that outcome; for missing baseline data we used an indicator variable as describe by White & Thompson,^22^ which means for each outcome participants are included even if they do not have a baseline measurement.

**Table S3: Sensitivity 1 intention to treat analyses of the effect of AFLY5 intervention on primary and secondary outcomes assessed immediately after the end of the intervention**. Numbers vary by outcome as indicated in the table.

| **Outcome** | **Control group (reference group)** | | **Intervention group** | | **Main effect (group difference)** | | |
| --- | --- | --- | --- | --- | --- | --- | --- |
|  | **Number** | **Mean (SD), median (IQR) or number (%)** | **Number** | **Mean (SD), median (IQR) or number (%)** | **Number** | **Difference in means or odds ratio (95%C)** | **p-value** |
| **Continuously measured outcomes** | | | | | | | |
| **Time spent in MVPA (minutes per day)** |  |  |  |  |  |  |  |
| **Time spent in SB (minutes per day)** |  |  |  |  |  |  |  |
| **Servings of fruit and vegetables (number per day)** |  |  |  |  |  |  |  |
| Time spent screen-viewing (minutes per day weekday) |  |  |  |  |  |  |  |
| Time spent screen-viewing (minutes per day Saturday) |  |  |  |  |  |  |  |
| BMI (z(sd)-score) |  |  |  |  |  |  |  |
| WC (z(sd)-score) |  |  |  |  |  |  |  |
| Servings of snacks (number per day) |  |  |  |  |  |  |  |
| Servings of high fat foods (number per day) |  |  |  |  |  |  |  |
| Servings of high energy drinks (number per day) |  |  |  |  |  |  |  |
| **Binary measured outcomes** | | | | | | | |
| Generally overweight/obese |  |  |  |  |  |  |  |
| Centrally overweight/obese |  |  |  |  |  |  |  |

Outcomes in bold are primary outcomes (p ≤ 0.05 indicates statistical significance); all others are secondary outcomes (p ≤ 0.05 after multiplying the actual p-value by 9 (to adjust for multiple testing of secondary outcomes) indicates statistical significance)

All differences in means / odds ratios with their 95%CI have been estimated using a multi-level model to account for clustering (non-independence) among children from the same school. Multi-level multivariable linear regression was used for effects of the intervention on continuously measured outcomes and multi-level multivariable logistic regression was used for binary outcomes.

The following baseline / school stratifying covariables were included: age, gender, the baseline measure of the outcome under consideration, school involvement in other health promoting behaviours, school area level deprivation.

MVPA: moderate and vigorous physical activity (accelerometer assessed), SB: sedentary behaviour (accelerometer assessed), BMI: body mass index, WC: waist circumference, F&V fruit and vegetables.

In these analyses participants were only included for each outcome if they had a baseline and a follow-up measurement of that outcome.**Table S4: Sensitivity 2 intention to treat analyses of the effect of AFLY5 intervention on primary and secondary outcomes assessed immediately after the end of the intervention**. Numbers included are identical for the three primary outcomes (N = xxx) but can vary by outcome for secondary outcomes (though none of these can be higher than xxx) as indicated in the table.

| **Outcome** | **Control group (reference group)** | | **Intervention group** | | **Main effect (group difference)** | | |
| --- | --- | --- | --- | --- | --- | --- | --- |
|  | **Number** | **Mean (SD), median (IQR) or number (%)** | **Number** | **Mean (SD), median (IQR) or number (%)** | **Number** | **Difference in means or odds ratio (95%C)** | **p-value** |
| **Continuously measured outcomes** | | | | | | | |
| **Time spent in MVPA (minutes per day)** |  |  |  |  |  |  |  |
| **Time spent in SB (minutes per day)** |  |  |  |  |  |  |  |
| **Servings of fruit and vegetables (number per day)** |  |  |  |  |  |  |  |
| Time spent screen-viewing (minutes per day weekday) |  |  |  |  |  |  |  |
| Time spent screen-viewing (minutes per day Saturday) |  |  |  |  |  |  |  |
| BMI (z(sd)-score) |  |  |  |  |  |  |  |
| WC (z(sd)-score) |  |  |  |  |  |  |  |
| Servings of snacks (number per day) |  |  |  |  |  |  |  |
| Servings of high fat foods (number per day) |  |  |  |  |  |  |  |
| Servings of high energy drinks (number per day) |  |  |  |  |  |  |  |
| **Binary measured outcomes** | | | | | | | |
| Generally overweight/obese |  |  |  |  |  |  |  |
| Centrally overweight/obese |  |  |  |  |  |  |  |

Outcomes in bold are primary outcomes (p ≤ 0.05 indicates statistical significance); all others are secondary outcomes (p ≤ 0.05 after multiplying the actual p-value by 9 (to adjust for multiple testing of secondary outcomes) indicates statistical significance)

All differences in means / odds ratios with their 95%CI have been estimated using a multi-level model to account for clustering (non-independence) among children from the same school. Multi-level multivariable linear regression was used for effects of the intervention on continuously measured outcomes and multi-level multivariable logistic regression was used for binary outcomes.

The following baseline / school stratifying covariables were included: age, gender, the baseline measure of the outcome under consideration, school involvement in other health promoting behaviours, school area level deprivation.

MVPA: moderate and vigorous physical activity (accelerometer assessed), SB: sedentary behaviour (accelerometer assessed), BMI: body mass index, WC: waist circumference, F&V fruit and vegetables.

In these analyses participants were only included for each outcome if they had a baseline and a follow-up measurement for all three primary outcomes. Numbers included are identical for the three primary outcomes (N = XXX) but can vary by outcome for secondary outcomes (though none of these can be higher than XXX) as indicated in the table. Missing baseline data for these secondary outcomes (once those with missing baseline primary outcomes are excluded) were managed as in the main analyses using the method described in.^22^

**Table S5: Sensitivity 3 intention to treat analyses of the effect of AFLY5 intervention on primary and secondary outcomes assessed immediately after the end of the intervention, with missing data for either baseline or follow-up measure of an outcome assumed to be 10% healthier than the average value in the study sample**. N = xxx

| **Outcome** | **Control group (reference group)** | | **Intervention group** | | **Main effect (group difference)** | | |
| --- | --- | --- | --- | --- | --- | --- | --- |
|  | **Number** | **Mean (SD), median (IQR) or number (%)** | **Number** | **Mean (SD), median (IQR) or number (%)** | **Number** | **Difference in means or odds ratio (95%C)** | **p-value** |
| **Continuously measured outcomes** | | | | | | | |
| **Time spent in MVPA (minutes per day)** |  |  |  |  |  |  |  |
| **Time spent in SB (minutes per day)** |  |  |  |  |  |  |  |
| **Servings of fruit and vegetables (number per day)** |  |  |  |  |  |  |  |
| Time spent screen-viewing (minutes per day weekday) |  |  |  |  |  |  |  |
| Time spent screen-viewing (minutes per day Saturday) |  |  |  |  |  |  |  |
| BMI (z(sd)-score) |  |  |  |  |  |  |  |
| WC (z(sd)-score) |  |  |  |  |  |  |  |
| Servings of snacks (number per day) |  |  |  |  |  |  |  |
| Servings of high fat foods (number per day) |  |  |  |  |  |  |  |
| Servings of high energy drinks (number per day) |  |  |  |  |  |  |  |
| **Binary measured outcomes** | | | | | | | |
| Generally overweight/obese |  |  |  |  |  |  |  |
| Centrally overweight/obese |  |  |  |  |  |  |  |

Outcomes in bold are primary outcomes (p ≤ 0.05 indicates statistical significance); all others are secondary outcomes (p ≤ 0.05 after multiplying the actual p-value by 9 (to adjust for multiple testing of secondary outcomes) indicates statistical significance)

All differences in means / odds ratios with their 95%CI have been estimated using a multi-level model to account for clustering (non-independence) among children from the same school. Multi-level multivariable linear regression was used for effects of the intervention on continuously measured outcomes and multi-level multivariable logistic regression was used for binary outcomes.

The following baseline / school stratifying covariables were included: age, gender, the baseline measure of the outcome under consideration, school involvement in other health promoting behaviours, school area level deprivation.

MVPA: moderate and vigorous physical activity (accelerometer assessed), SB: sedentary behaviour (accelerometer assessed), BMI: body mass index, WC: waist circumference, F&V fruit and vegetables.

In these analyses participants all participants are included (N = xxx (the number of participants recruited to the study). Missing baseline data is managed as in the main effectiveness analysis (Table S2) and as described in^22^ and missing outcome data are imputed on the basis of those with missing data being 10% healthier than the average (mean or median) for all participants in the study for a given outcome.

**Table S6: Sensitivity 4 intention to treat analyses of the effect of AFLY5 intervention on primary and secondary outcomes assessed immediately after the end of the intervention, with missing data for either baseline or follow-up measure of an outcome assumed to be 10% less healthy than the average value in the study sample**. N = xxx

| **Outcome** | **Control group (reference group)** | | **Intervention group** | | **Main effect (group difference)** | | |
| --- | --- | --- | --- | --- | --- | --- | --- |
|  | **Number** | **Mean (SD), median (IQR) or number (%)** | **Number** | **Mean (SD), median (IQR) or number (%)** | **Number** | **Difference in means or odds ratio (95%C)** | **p-value** |
| **Continuously measured outcomes** | | | | | | | |
| **Time spent in MVPA (minutes per day)** |  |  |  |  |  |  |  |
| **Time spent in SB (minutes per day)** |  |  |  |  |  |  |  |
| **Servings of fruit and vegetables (number per day)** |  |  |  |  |  |  |  |
| Time spent screen-viewing (minutes per day weekday) |  |  |  |  |  |  |  |
| Time spent screen-viewing (minutes per day Saturday) |  |  |  |  |  |  |  |
| BMI (z(sd)-score) |  |  |  |  |  |  |  |
| WC (z(sd)-score) |  |  |  |  |  |  |  |
| Servings of snacks (number per day) |  |  |  |  |  |  |  |
| Servings of high fat foods (number per day) |  |  |  |  |  |  |  |
| Servings of high energy drinks (number per day) |  |  |  |  |  |  |  |
| **Binary measured outcomes** | | | | | | | |
| Generally overweight/obese |  |  |  |  |  |  |  |
| Centrally overweight/obese |  |  |  |  |  |  |  |

Outcomes in bold are primary outcomes (p ≤ 0.05 indicates statistical significance); all others are secondary outcomes (p ≤ 0.05 after multiplying the actual p-value by 9 (to adjust for multiple testing of secondary outcomes) indicates statistical significance)

All differences in means / odds ratios with their 95%CI have been estimated using a multi-level model to account for clustering (non-independence) among children from the same school. Multi-level multivariable linear regression was used for effects of the intervention on continuously measured outcomes and multi-level multivariable logistic regression was used for binary outcomes.

The following baseline / school stratifying covariables were included: age, gender, the baseline measure of the outcome under consideration, school involvement in other health promoting behaviours, school area level deprivation.

MVPA: moderate and vigorous physical activity (accelerometer assessed), SB: sedentary behaviour (accelerometer assessed), BMI: body mass index, WC: waist circumference, F&V fruit and vegetables.

In these analyses participants all participants are included (N = xxx (the number of participants recruited to the study). Missing baseline data is managed as in the main table and as described in^22^ and missing outcome data are imputed on the basis of those with missing data being 10% healthier than all participants in the study for a given outcome.

In these analyses participants all participants are included (N = xxx (the number of participants recruited to the study). Missing baseline data is managed as in the main effectiveness analysis (Table S2) and as described in^22^ and missing outcome data are imputed on the basis of those with missing data being 10% less healthy than the average (mean or median) for all participants in the study for a given outcome.

**Table S7a: Per-protocol analyses of the effect of AFLY5 intervention on primary and secondary outcomes assessed immediately after the end of the intervention**. Numbers vary by outcome as indicated in the table.

| **Outcome** | **Control group (reference group)** | | **Intervention group** | | **Main effect (group difference)** | | |
| --- | --- | --- | --- | --- | --- | --- | --- |
|  | **Number** | **Mean (SD), median (IQR) or number (%)** | **Number** | **Mean (SD), median (IQR) or number (%)** | **Number** | **Difference in means or odds ratio (95%C)** | **p-value** |
| **Continuously measured outcomes** | | | | | | | |
| **Time spent in MVPA (minutes per day)** |  |  |  |  |  |  |  |
| **Time spent in SB (minutes per day)** |  |  |  |  |  |  |  |
| **Servings of fruit and vegetables (number per day)** |  |  |  |  |  |  |  |
| Time spent screen-viewing (minutes per day weekday) |  |  |  |  |  |  |  |
| Time spent screen-viewing (minutes per day Saturday) |  |  |  |  |  |  |  |
| BMI (z(sd)-score) |  |  |  |  |  |  |  |
| WC (z(sd)-score) |  |  |  |  |  |  |  |
| Servings of snacks (number per day) |  |  |  |  |  |  |  |
| Servings of high fat foods (number per day) |  |  |  |  |  |  |  |
| Servings of high energy drinks (number per day) |  |  |  |  |  |  |  |
| **Binary measured outcomes** | | | | | | | |
| Generally overweight/obese |  |  |  |  |  |  |  |
| Centrally overweight/obese |  |  |  |  |  |  |  |

Per-protocol analysis defined as teaching at least 70% (11 out of the 16) AFLY5 lessons. All participants from the intervention schools where the teacher log indicates that they have taught fewer than 11 lessons are excluded from these analyses. In these analyses (Table S7a) if the teacher had not returned their log and hence the number of lessons that they had taught is unknown we have assumed that they taught 11 or more lessons (pupils included).

Outcomes in bold are primary outcomes (p ≤ 0.05 indicates statistical significance); all others are secondary outcomes (p ≤ 0.05 after multiplying the actual p-value by 9 (to adjust for multiple testing of secondary outcomes) indicates statistical significance)

All differences in means / odds ratios with their 95%CI have been estimated using a multi-level model to account for clustering (non-independence) among children from the same school. Multi-level multivariable linear regression was used for effects of the intervention on continuously measured outcomes and multi-level multivariable logistic regression was used for binary outcomes.

The following baseline / school stratifying covariables were included: age, gender, the baseline measure of the outcome under consideration, school involvement in other health promoting behaviours, school area level deprivation.

MVPA: moderate and vigorous physical activity (accelerometer assessed), SB: sedentary behaviour (accelerometer assessed), BMI: body mass index, WC: waist circumference, F&V fruit and vegetables.

In these analyses, after removal of schools that did not teach at least 11 out of 16 of the lessons, participants were only included for each outcome if they had a follow-up measurement of that outcome. For partial missing baseline data we used an indicator variable as describe by White & Thompson,^22^ which means for each outcome participants are included even if they do not have a baseline measurement.

**Table S7b: Per-protocol analyses of the effect of AFLY5 intervention on primary and secondary outcomes assessed immediately after the end of the intervention**. Numbers vary by outcome as indicated in the table.

| **Outcome** | **Control group (reference group)** | | **Intervention group** | | **Main effect (group difference)** | | |
| --- | --- | --- | --- | --- | --- | --- | --- |
|  | **Number** | **Mean (SD), median (IQR) or number (%)** | **Number** | **Mean (SD), median (IQR) or number (%)** | **Number** | **Difference in means or odds ratio (95%C)** | **p-value** |
| **Continuously measured outcomes** | | | | | | | |
| **Time spent in MVPA (minutes per day)** |  |  |  |  |  |  |  |
| **Time spent in SB (minutes per day)** |  |  |  |  |  |  |  |
| **Servings of fruit and vegetables (number per day)** |  |  |  |  |  |  |  |
| Time spent screen-viewing (minutes per day weekday) |  |  |  |  |  |  |  |
| Time spent screen-viewing (minutes per day Saturday) |  |  |  |  |  |  |  |
| BMI (z(sd)-score) |  |  |  |  |  |  |  |
| WC (z(sd)-score) |  |  |  |  |  |  |  |
| Servings of snacks (number per day) |  |  |  |  |  |  |  |
| Servings of high fat foods (number per day) |  |  |  |  |  |  |  |
| Servings of high energy drinks (number per day) |  |  |  |  |  |  |  |
| **Binary measured outcomes** | | | | | | | |
| Generally overweight/obese |  |  |  |  |  |  |  |
| Centrally overweight/obese |  |  |  |  |  |  |  |

Per-protocol analysis defined as teaching at least 70% (11 out of the 16) AFLY5 lessons. All participants from the intervention schools where the teacher log indicates that they have taught fewer than 11 lessons are excluded from these analyses. In these analyses (Table S7b) if the teacher had not returned their log and hence the number of lessons that they had taught is unknown we have assumed that they did not teach 11 or more lessons (pupils excluded).

Outcomes in bold are primary outcomes (p ≤ 0.05 indicates statistical significance); all others are secondary outcomes (p ≤ 0.05 after multiplying the actual p-value by 9 (to adjust for multiple testing of secondary outcomes) indicates statistical significance)

All differences in means / odds ratios with their 95%CI have been estimated using a multi-level model to account for clustering (non-independence) among children from the same school. Multi-level multivariable linear regression was used for effects of the intervention on continuously measured outcomes and multi-level multivariable logistic regression was used for binary outcomes.

The following baseline / school stratifying covariables were included: age, gender, the baseline measure of the outcome under consideration, school involvement in other health promoting behaviours, school area level deprivation.

MVPA: moderate and vigorous physical activity (accelerometer assessed), SB: sedentary behaviour (accelerometer assessed), BMI: body mass index, WC: waist circumference, F&V fruit and vegetables.

In these analyses, after removal of schools that did not teach at least 11 out of 16 of the lessons, participants were only included for each outcome if they had a follow-up measurement of that outcome. For partial missing baseline data we used an indicator variable as describe by White & Thompson,^22^ which means for each outcome participants are included even if they do not have a baseline measurement.**Table S8: Main analyses of the effect of AFLY5 intervention on primary and secondary outcomes assessed immediately after the end of the intervention stratified by gender**. Numbers vary by outcome as indicated in the table.

|  | **Outcome** | **Control group (reference group)** | | **Intervention group** | | **Main effect (group difference)** | | |
| --- | --- | --- | --- | --- | --- | --- | --- | --- |
|  |  | **Number** | **Mean (SD), median (IQR) or number (%)** | **Number** | **Mean (SD), median (IQR) or number (%)** | **Number** | **Difference in means or odds ratio (95%C)** | **Interaction coefficient (95%CI)**  **[p-value interaction]*** |
| **Continuously measured outcomes** | | | | | | | | |
| **MVPA** | Female |  |  |  |  |  |  |  |
|  | Male |  |  |  |  |  |  |  |
| **SB** | Female |  |  |  |  |  |  |  |
|  | Male |  |  |  |  |  |  |  |
| **F&V** | Female |  |  |  |  |  |  |  |
|  | Male |  |  |  |  |  |  |  |
| Snacks | Female |  |  |  |  |  |  |  |
|  | Male |  |  |  |  |  |  |  |
| High fat | Female |  |  |  |  |  |  |  |
|  | Male |  |  |  |  |  |  |  |
| High energy drink | Female |  |  |  |  |  |  |  |
|  | Male |  |  |  |  |  |  |  |
| SV weekday | Female |  |  |  |  |  |  |  |
|  | Male |  |  |  |  |  |  |  |
| SV Saturday | Female |  |  |  |  |  |  |  |
|  | Male |  |  |  |  |  |  |  |
| BMI | Female |  |  |  |  |  |  |  |
|  | Male |  |  |  |  |  |  |  |
| WC | Female |  |  |  |  |  |  |  |
|  | Male |  |  |  |  |  |  |  |
| **Binary measured outcomes** | | | | | | | | |
| General overweight | Female |  |  |  |  |  |  |  |
|  | Male |  |  |  |  |  |  |  |
| Central overweight | Female |  |  |  |  |  |  |  |
|  | Male |  |  |  |  |  |  |  |

Outcomes in bold are primary outcomes (p ≤ 0.05 indicates statistical significance); all others are secondary outcomes (p ≤ 0.05 after multiplying the actual p-value by 9 (to adjust for multiple testing of secondary outcomes) indicates statistical significance)

All differences in means / odds ratios with their 95%CI have been estimated using a multi-level model to account for clustering (non-independence) among children from the same school. Multi-level multivariable linear regression was used for effects of the intervention on continuously measured outcomes and multi-level multivariable logistic regression was used for binary outcomes.

The following baseline / school stratifying covariables were included: age, gender, the baseline measure of the outcome under consideration, school involvement in other health promoting behaviours, school area level deprivation.

MVPA: moderate and vigorous physical activity (accelerometer assessed), SB: sedentary behaviour (accelerometer assessed), F&V fruit and vegetables, SV: Screen viewing, BMI: body mass index, WC: waist circumference.

In these analyses, after removal of schools that did not teach at least 11 out of 16 of the lessons, participants were only included for each outcome if they had a follow-up measurement of that outcome. For partial missing baseline data we used an indicator variable as describe by White & Thompson,^22^ which means for each outcome participants are included even if they do not have a baseline measurement.

* Giving the magnitude and 95% CI for the difference in effects between females and males and p-value testing the null hypothesis that the effect is the same in females and males

**Table S9: Main analyses of the effect of AFLY5 intervention on primary and secondary outcomes assessed immediately after the end of the intervention stratified by school deprivation**. Numbers vary by outcome as indicated in the table.

|  | **Outcome** | **Control group (reference group)** | | **Intervention group** | | **Main effect (group difference)** | | |
| --- | --- | --- | --- | --- | --- | --- | --- | --- |
|  |  | **Number** | **Mean (SD), median (IQR) or number (%)** | **Number** | **Mean (SD), median (IQR) or number (%)** | **Number** | **Difference in means or odds ratio (95%C)** | **Interaction coefficient (95%CI)**  **[p-value interaction]*** |
| **Continuously measured outcomes** | | | | | | | | |
| **MVPA** | Low |  |  |  |  |  |  |  |
|  | Medium |  |  |  |  |  |  |  |
|  | High |  |  |  |  |  |  |  |
| **SB** | Low |  |  |  |  |  |  |  |
|  | Medium |  |  |  |  |  |  |  |
|  | High |  |  |  |  |  |  |  |
| **F&V** | Low |  |  |  |  |  |  |  |
|  | Medium |  |  |  |  |  |  |  |
|  | High |  |  |  |  |  |  |  |
| Snacks | Low |  |  |  |  |  |  |  |
|  | Medium |  |  |  |  |  |  |  |
|  | High |  |  |  |  |  |  |  |
| High fat | Low |  |  |  |  |  |  |  |
|  | Medium |  |  |  |  |  |  |  |
|  | High |  |  |  |  |  |  |  |
| High energy drink | Low |  |  |  |  |  |  |  |
|  | Medium |  |  |  |  |  |  |  |
|  | High |  |  |  |  |  |  |  |
| SV weekday | Low |  |  |  |  |  |  |  |
|  | Medium |  |  |  |  |  |  |  |
|  | High |  |  |  |  |  |  |  |
| SV Saturday | Low |  |  |  |  |  |  |  |
|  | Medium |  |  |  |  |  |  |  |
|  | High |  |  |  |  |  |  |  |
| BMI | Low |  |  |  |  |  |  |  |
|  | Medium |  |  |  |  |  |  |  |
|  | High |  |  |  |  |  |  |  |
| WC | Low |  |  |  |  |  |  |  |
|  | Medium |  |  |  |  |  |  |  |
|  | High |  |  |  |  |  |  |  |
| **Binary measured outcomes** | | | | | | | | |
| General overweight | Low |  |  |  |  |  |  |  |
|  | Medium |  |  |  |  |  |  |  |
|  | High |  |  |  |  |  |  |  |
| Central overweight | Low |  |  |  |  |  |  |  |
|  | Medium |  |  |  |  |  |  |  |
|  | High |  |  |  |  |  |  |  |

Outcomes in bold are primary outcomes (p ≤ 0.05 indicates statistical significance); all others are secondary outcomes (p ≤ 0.05 after multiplying the actual p-value by 9 (to adjust for multiple testing of secondary outcomes) indicates statistical significance)

All differences in means / odds ratios with their 95%CI have been estimated using a multi-level model to account for clustering (non-independence) among children from the same school. Multi-level multivariable linear regression was used for effects of the intervention on continuously measured outcomes and multi-level multivariable logistic regression was used for binary outcomes.

The following baseline / school stratifying covariables were included: age, gender, the baseline measure of the outcome under consideration, school involvement in other health promoting behaviours, school area level deprivation.

MVPA: moderate and vigorous physical activity (accelerometer assessed), SB: sedentary behaviour (accelerometer assessed), F&V fruit and vegetables, SV: Screen viewing, BMI: body mass index, WC: waist circumference.

In these analyses, after removal of schools that did not teach at least 11 out of 16 of the lessons, participants were only included for each outcome if they had a follow-up measurement of that outcome. For partial missing baseline data we used an indicator variable as describe by White & Thompson,^22^ which means for each outcome participants are included even if they do not have a baseline measurement.

* Giving the magnitude and 95% CI for the difference in effects between low and medium deprivation and between low and high deprivation and the p-value testing the null hypothesis that the effect is the same in each third of deprivation.

**Table S10: Secondary intention to treat analyses of the effect of AFLY5 intervention on potential mediators assessed immediately after the end of the intervention**. Numbers vary by outcome (mediator) as indicated in the table.

| **Mediator (outcome in these analyses)** | **Control group (reference group)** | | **Intervention group** | | **Main effect (group difference)** | | |
| --- | --- | --- | --- | --- | --- | --- | --- |
|  | **Number** | **Mean (SD) or median (IQR)** | **Number** | **Mean (SD) or median (IQR)** | **Number** | **Difference in means (95%C)** | **p-value** |
| Self efficacy for PA |  |  |  |  |  |  |  |
| Self efficacy for F&V |  |  |  |  |  |  |  |
| Maternal modelling PA |  |  |  |  |  |  |  |
| Paternal modelling PA |  |  |  |  |  |  |  |
| Maternal support for PA |  |  |  |  |  |  |  |
| Paternal support for PA |  |  |  |  |  |  |  |
| Maternal restriction of SB |  |  |  |  |  |  |  |
| Paternal restriction of SB |  |  |  |  |  |  |  |
| Parental modelling of F&V |  |  |  |  |  |  |  |
| Child’s knowledge |  |  |  |  |  |  |  |

In these analyses the outcomes are potential mediators of the effect on the three main outcomes (p ≤ 0.05 after multiplying the actual p-value by 10 (to adjust for multiple testing of mediators) indicates statistical significance).

All differences in means with their 95%CI have been estimated using a multi-level linear regression model to account for clustering (non-independence) among children from the same school.

The following baseline / school stratifying covariables were included: age, gender, the baseline measure of the outcome under consideration, school involvement in other health promoting behaviours, school area level deprivation.

PA: physical activity, SB: sedentary behaviour, F&V fruit and vegetables, SV: Screen viewing.

In these analyses, participants were only included for each mediator (here treated as an outcome) if they had a follow-up measurement of that mediator. For partial missing baseline data we used an indicator variable as describe by White & Thompson,^22^ which means for each mediator participants are included even if they do not have a baseline measurement. NB: for knowledge there was no baseline measurement and so only those with an outcome measurement are included here.

**Table S11: Secondary intention to treat analysis effect of AFLY5 intervention on primary outcomes with adjustment for potential mediators.** N varies for each outcome.

| **Outcome** | **Mediator** | **Main effect without mediator adjustment** | | **Mediator effect with mediator adjustment** | | **% change with addition of mediator** |
| --- | --- | --- | --- | --- | --- | --- |
|  |  | **Number** | **Difference in means or OR (95% CI)** | **Number** | **Difference in means or OR (95% CI)** |  |
| MVPS | M1* |  |  |  |  |  |
|  | M2* |  |  |  |  |  |
|  | M3* |  |  |  |  |  |
|  | M4* |  |  |  |  |  |
|  | Mall* |  |  |  |  |  |
| SB | M1* |  |  |  |  |  |
|  | M2* |  |  |  |  |  |
|  | M3* |  |  |  |  |  |
|  | M4* |  |  |  |  |  |
|  | Mall* |  |  |  |  |  |
| F&V | M1* |  |  |  |  |  |
|  | M2* |  |  |  |  |  |
|  | M3* |  |  |  |  |  |
|  | Mall* |  |  |  |  |  |

All differences in means with their 95%CI have been estimated using a multi-level linear model to account for clustering (non-independence) among children from the same school.

MVPA: moderate and vigorous physical activity (accelerometer assessed), SB: sedentary behaviour (accelerometer assessed), F&V fruit and vegetables.

In these analyses participants were only included for each outcome if they had a follow-up measurement of that outcome and of any relevant mediator that will be added to the model. For partial missing baseline data on outcome or mediators we used an indicator variable as describe by White & Thompson,^22^ which means for each outcome participants are included even if they do not have a baseline measurement.

* See section 2.3 above for criteria for these analyses (they will only be done where the intervention has had an effect on one of the 3 primary outcomes and has also had an effect on at least one of the mediators relevant to that outcome). For all mediators/ situations that fulfil these criteria their mediation effect will be assessed separately (indicated by M1, M2, ...) and then with all of them included together in a model (Mall).

**Table S12: Long-term effectiveness analysis: effect of intervention on change in outcome between baseline and long-term follow-up.** N varies for each outcome.

| **Outcome** | **Number** | **Difference in change means or odds comparing pupils from intervention to those from control schools (95% CI)** | | | **p-interaction with time*** |
| --- | --- | --- | --- | --- | --- |
|  |  | **Between baseline and immediate follow-up** | **Between immediate and long-term follow-up** | **Between baseline and long-term follow-up** |  |
| **Time spent in MVPA (minutes per day)** |  |  |  |  |  |
| **Time spent in SB (minutes per day)** |  |  |  |  |  |
| **Servings of fruit and vegetables (number per day)** |  |  |  |  |  |
| Time spent screen-viewing (minutes per day weekday) |  |  |  |  |  |
| Time spent screen-viewing (minutes per day Saturday) |  |  |  |  |  |
| BMI (z(sd)-score) |  |  |  |  |  |
| WC (z(sd)-score) |  |  |  |  |  |
| Servings of snacks (number per day) |  |  |  |  |  |
| Servings of high fat foods (number per day) |  |  |  |  |  |
| Servings of high energy drinks (number per day) |  |  |  |  |  |
| **Binary outcomes** | | | | | |
| General overweight |  |  |  |  |  |
| Central overweight |  |  |  |  |  |

Outcomes in bold are primary outcomes (p ≤ 0.05 indicates statistical significance); all others are secondary outcomes (p ≤ 0.05 after multiplying the actual p-value by 9 (to adjust for multiple testing of secondary outcomes) indicates statistical significance)

All differences in change in means / odds ratios with their 95%CI have been estimated using a multi-level model to account for clustering (non-independence) among children from the same school and to account for repeat measurements within children. Multi-level multivariable linear regression was used for effects of the intervention on continuously measured outcomes and multi-level multivariable logistic regression was used for binary outcomes.

The following baseline / school stratifying covariables were included: age, gender, the baseline measure of the outcome under consideration, school involvement in other health promoting behaviours, school area level deprivation.

MVPA: moderate and vigorous physical activity (accelerometer assessed), SB: sedentary behaviour (accelerometer assessed), BMI: body mass index, WC: waist circumference, F&V fruit and vegetables.

In these analyses participants were included for each outcome if they had any one of a baseline, immediate or long-term follow-up measurement of that outcome.
