# Supplementary material for: The Active for Life Year 5 (AFLY5) school-based cluster randomised controlled trial protocol: detailed statistical analysis plan
Source: Trials. 2013 Jul 24;14:234. doi: 10.1186/1745-6215-14-234 (PMC3733690; doi:10.1186/1745-6215-14-234)
Supplement: Additional file 2 — AFLY5 Diet Data Coding in Section A of Questionnaire. Food and drink items for diet categories. [file 1745-6215-14-234-S2.doc]

**Additional file 2**

# AFLY5 Diet Data Coding in Section A of Questionnaire

**Food and drink items for diet categories**

**Fruit - ‘F’**

Fruit

Stewed fruit

Tinned fruit

Dried fruit

Sweet potato

Cereal with fruit

Fruit tuck/Fruit pack

Multiples of smaller fruit e.g. grapes, 2 plums, half a banana

Fruit salads

**Vegetables - ‘V’**

Vegetables

Tinned vegetables

Dried vegetables

Sweet potato

Tomato

Swede

Parsnip

Pulses: maximum of one score per day

Salads

**Savoury snacks – ‘Sa Sn’**

Potato crisps, corn chips

Manufactured savoury snacks

Dry or savoury biscuits, crispbread

Nuts

Pepperami

Popcorn

Pretzels

Crackers when not part of a meal

**Sweet snacks – ‘Sw Sn’**

Cake

Muffins

Scones

Croissants

Biscuits

Muesli bars

Chocolate

Chocolate bars

Sweets

Other confectionary

Ice confectionary (including ice cream and ice pops)

Puddings

Sweet pies or pastry

Dairy desserts

Custard

Hot cross buns

Malf loaf

Poptart

Pain au chocolat

Jelly

# High energy savoury convenience foods – ‘HFF’

Chips

Fries

Potato wedges

Hashbrowns

Smiles

Sausage/Veggie sausage

Burger

Chicken, fish and turkey coated food e.g. nuggets, fishfingers, chicken dippers

KFC

MacDonalds

Veggiburger

Meat pastries (e.g pasty)

Meat pies (e.g. pork pie)

Kebabs

Pizza

Scampi

Bacon

Fish cakes

Scotch eggs

Sausage roll

Fritters

Hotdog

Hot pocket

Pancake

Waffle (potato or sweet type)

Smilies

Cornbeef

Dairylee dunker

**Other food – ‘Oth’**

Cereals (not cereal bars or Coco straws)

Bread

Rolls

Toast

Sandwich

Pitta

Wraps

Crumpet

Brioche

Savoury pancakes

Cous cous

Rice

Pasta (including alphabet letters)

Potato (not chips, wedges or smilies)

Yorkshire pudding

Yogurt - fruit or vanilla (including frube) Not toffee or chocolate yoghurts.

Cheese (including cheese string)

Egg

Homous

Quorn

Lentils

Meat (not chicken nuggets or sausages)

Fish (not fishfingers)

Coleslaw

Onion bhajis

Vegetable samosas

Soup

Stuffing

Composite foods scored as one eg. Ham roll

Composite meals eg. lasagne, curry

Dips

Quiche

Flan

Pot noodle

Non specific items (including ‘food’, ‘breakfast’, ‘pack lunch’, ‘school dinners’, ‘indian’, ‘chinese’, ‘snack’, ‘BBQ’)

Illegible food items

**High-energy fluids – ‘HE dri’**

Fruit juice

Fruit juice drink

Cordial

Flavoured mineral water

Carbonated and still soft drinks

Smoothies

Chocolate drinks

Yoghurt drinks

Milkshakes

## Other drinks – ‘Oth dri’

Water

Milk (including milk with honey)

Tea

Coffee

Low calorie or diet drinks
Illegible drink items

Non specific items (‘yes’, ‘drink’ ‘🗸’ etc)

When a child has used ‘or’, code High Energy if HE drink present, or if not code Other (only score 1 total)

## NOTES

## Items not coded

Sandwich fillings e.g. ham, bacon, sausage, cheese, egg, jam

Items added to toast e.g. butter, jam, honey

Sauces and dressings eg. Tomato ketchup, mayonnaise

Medicines and vitamins

Chewing gum

**fruit and vegetables**

**Sauces/curry/soup/pies etc**

Do not record tomato sauce /ketchup as vegetable

Do not score any veg listed as an ingredient to a sauce/curry/soup/pie/dish etc.

E.g. Pasta with tuna sweetcorn sauce = 0

Pasta with tuna and sweetcorn = 1

Chicken & asparagus pie = 0

Chicken pie & asparagus = 1

Pasta tomato sauce = 0

Tomato pasta = 1

Leek & potato soup = 0

Sweet potato curry = 0

Vegetable lasagne = 0

**Pizza topping/ Sandwich filling/ Jacket potato filling**

Do not score any item listed as a pizza topping, sandwich or jacket potato filling

E.g. Ham and tomato sandwich = 0

Ham sandwich, tomato = 1

Pizza with peppers, chicken and sweetcorn = 0

**Salads/Fruit Salads**

If word ‘salad’ is not mentioned, each item scores 1. If ‘salad’ is mentioned, 1 total.

E.g. Tomato, lettuce, peppers = 3

Salad (tomato, lettuce, peppers) = 1

Fruit salad, grapes, pineapple, orange = 1

Grapes, pineapple, orange = 3

Where by ‘salad’ they mean leaves/lettuce, score 1

E.g. Tomato, salad, cucumber = 3

**Fruit puddings**

Whole fruit as part of a pudding scores 1, fruits that are not in natural/whole form in a pudding.do not score

E.g. Apple crumble/pie = 0

Apple with natural yoghurt = 1

Strawberries & meringue = 1

Icecream & strawberries = 1

Strawberry cheesecake = 0

**Fruit & Veg general rules**

“Potato” is not a vegetable it is ‘other food’

“Pulses” (beans, baked beans, chick peas) are given maximum of one score per day as a vegetable; if they eat it twice, ignore the second record.

When child lists “vegetables” along with other named items of vegetable, do not score as additional item. When just “vegetables” listed (i.e. no named items), score 1.

E.g. Vegetables, carrots, peas = 2

Vegetables = 1

When child lists “fruit” along with other named items of fruit, do not score as additional item. When just “fruit” listed (i.e. no named items), score 1.

E.g. Fruit, banana = 1

Fruit = 1

“Salad bar” score as ‘1’

Cereal with fruit (fresh or dried) score as ‘1’ E.g. Fruit & Fibre, Fruit & Nut Muesli, Branflakes with banana

“Fruit bars” – do not code as fruit, code as ‘sweet snack’.

Fruits written before an item which may be fruit flavoured should NOT be scored unless a comma is present:

Banana cake = 0, but Banana, cake = 1. Orange frube = 0, but Orange, frube = 1

Fruit yoghurt = 0, but Fruit, yoghurt = 1. Peach yoghurt = 0, but Peach, yoghurt = 1

**OTHER FOOD**

“Potato” is not recorded as a vegetable it is recorded as ‘other food’

“Toffee and chocolate yogurts” are recorded as a sweet food, but other yogurts are recorded as ‘other food’

Foods which are “combined” eg curry, pizza or lasagne, even if they are vegetarian, record as an ‘other food’ and not a vegetable.

**DRINKS**

If the child has written a drink under the answer to a question about food, it will be scored in the drink form, but the text will only show for the drink questions. If there is a score but no drink, look across at the food questions to check if the text about the drink is in the food question. If it is, confirm the score for the drink even though no text shows in the drink text.

**SAVOURY SNACKS**

“Lunchables” are a type of cracker.

**HIGH FAT FOODS**

“Smilies “(potatoes in smiley faces)

Any “nuggets”

Any “wedges”

“Pancakes” and “waffles” are high fat food, not sweet snacks

**SWEET SNACKS**

**The following are not fruits:**

“Fruit-winder”

“Fruit-flakes”

“Fruit bar”

**Duplicate scoring**

Sometimes food items will be given a double score e.g. “chocolate biscuit” will be automatically scored 1 for chocolate and 1 for biscuit; this needs changing to a score of 1.

Often drinks are given a double score which needs changing to 1 e.g. apple juice, because apple and juice will both be given a score of 1 because children also write ‘apple’ or just ‘juice.

**Repeated/multiple items**

Where items are repeated at the same time point, there is a maximum score of 1.

E.g. Lunch: 2 Oranges = 1

Break: Orange, Lunch: Orange = 1 for each

**Repeated text**

When the text is repeated in the question about after school and evening meal only code for evening meal (this is because before they turn the page they think the question is about the evening meal).

**Complete**

Mark as incomplete (on first tab) if child indicated they were not in school for part of the day or if more than half of the questions are incomplete or there is no text for two of the three main meals. If a child has written ‘no’ in a box, this does not count as incomplete.

Examples

|  |  | Fruit score | Veg score |
| --- | --- | --- | --- |
| 11001 | “Fruit apples banana grapes”. | 3 | 0 |
| 11014 | “vegetarian sausages, onion baghes and vegetable samosas”. | 0 | 0 |
| 11019 | “Oranges”. | 1 | 0 |
| 11025 | “Grapes, melon, apple” at breaktime | 3 | 0 |
| 12002 | “I had chicken casserol with carots and vedges”. | 0 | 1 |
| 13005 | “Pasta with meay and a tomato sause”. | 0 | 0 |
| 13016 | “fruit winder”. | 0 | 0 |
| 13007 | “fruit winder”. | 0 | 0 |
| 13018 | “I had tuna, noodles and sparagouse”. | 0 | 1 |
| 13024 | “Fruit (apples)”. | 1 | 0 |
| 13032 | “Meatballs and gravy, also vegetables”. | 0 | 1 |
| 13034 | “Chees and tommato pasta” | 0 | 1 |
| 14002 | “Beans, sausage and tomatos pasta” | 0 | 2 |
| 14004 | “Ice cream (strawberry)” | 0 | 0 |
| 14005 | “lemon custard” | 0 | 0 |
| 14009 | “Pizza, cheese and tomoto” | 0 | 1 |
| 14012 | “Fruit tack” | 1 | 0 |
| 14013 | “Roast beef - veg - inclooding parsnips - yorkshire puddings” | 0 | 1 |
| 14019 | “No! I had a black currant” between end of school and evening meal. | 0 | 0 |
| 15006 | “Blackcurrant jelly”. | 0 | 0 |
| 15018 | “vedibales”. | 0 | 1 |
| 15019 | “Stue (vegetable) pork”. | 0 | 0 |
| 15021 | “A peas of frout” | 1 | 0 |
| 15028 | “Cheese, onion and lettuce sandwiches, half an apple, winder” | 1 | 0 |
| 15040 | “I had pitta bread in the pitta I had cheese and tomato sauce and a scotch egg and a choclat bar” | 0 | 0 |
| 15041 | “Trifel, starberry and cherrie” | 2 | 0 |
| 15042 | “Apple and par apple pie” | 0 | 0 |
| 15043 | “fruit flakes” | 0 | 0 |
| 16002 | “I had a blackcurrant before I went to bed”. | 0 | 0 |
| 16018 | “Pasta with tomato” | 0 | 1 |
| 16028 | “Strawberry Angel Delight” | 0 | 0 |
| 18002 | “Sandwich, fruit bar, crisp”. | 0 | 0 |
| 18005 | “Vegetable lasagne” | 0 | 0 |
| 18018 | “Chocolate, cheese and biscuit banana, banana.” | 1 | 0 |
| 18019 | “Vegetarian cottage pie, peas and broccoli”. | 0 | 2 |
| 18024 | “I had packet of crisps, apple, cream egg, Maltesers, Milky Way, apple”. | 1 | 0 |
| 18052 | “I had a packet of fruit flakes” | 0 | 0 |
| 20002 | “Crispy pancakes chips and beans” repeated for ‘between school and evening meal and for evening meal. | 0 | 1 |
| 21016 | “Spinach and ricotta pasta with a tomato and basil sauce and sweetcorn”. | 0 | 2 |
| 22018 | “Two bowls of apples” for tuck. | 1 | 0 |
| 22022 | “Fruit salad jam tart apple. Brown bread ham sandwich.” | 2 | 0 |
| 24006 | “Chocolate, apple, chips, colslaw” for after school and “chips, colslaw” for evening meal. | 1 | 0 |
| 24025 | “Carrot cake”. | 0 | 0 |
| 25019 | “Kiwi x 3”. | 1 | 0 |
| 27006 | “Apple, banna and banana”.. | 2 | 0 |
| 27025 | “Cooked potatoes, brocley, carrots and pork chops” | 0 | 2 |
| 28009 | “I had fruit n Fiber” not coded for fruit | 1 | 0 |
| 28017 | “I had can of straberrys” | 1 | 0 |
| 28019 | After school “For my evening meal sausages chips and beans” and for dinner “Sausages chips and beans”. | 0 | 1 |
| 28020 | For breakfast: “I had cornflakes 8 apples and 4 bananas”.. | 2 | 0 |
| 14013 | “Cornflakes and sultanas with half spoonful of sugar milk semi-skimmed” | 1 | 0 |
| 15005 | “Fruit cocktail trifle” | 0 | 0 |
| 19016 | Breakfast: “Orange penguin”. | 0 | 0 |
| 21008 | Raison cake. | 0 | 0 |
| 27017 | “Sandwich and crispos fruit flacks chocolate bar and a frub (yougert)” | 0 | 0 |
