# Supplementary material for: The Active for Life Year 5 (AFLY5) school-based cluster randomised controlled trial protocol: detailed statistical analysis plan
Source: Trials. 2013 Jul 24;14:234. doi: 10.1186/1745-6215-14-234 (PMC3733690; doi:10.1186/1745-6215-14-234)
Supplement: Additional file 3 — Knowledge assessment devised by study team. [file 1745-6215-14-234-S3.doc]

**Additional file 3: Knowledge assessment devised by study team**


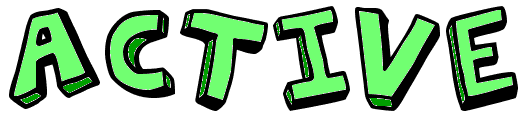

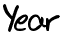

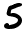

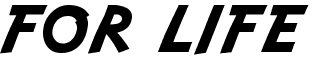


*Office use only – ID label here*

*Office use only – name label here*

**
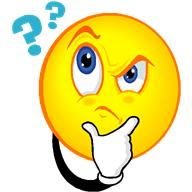
**

**SECTION G**

***Quick Quiz***

**Tick ONE answer for each of the questions below**.

1. How much physical activity do you think children should be doing each day to stay healthy?

| At least 15 minutes | At least 30 minutes | At least 60 minutes |
| --- | --- | --- |
|  |  |  |

1. People who watch TV all day are sometimes called ‘Couch potatoes’! For children to stay healthy, how much time can they spend each day, doing things like watching TV and playing computer games?

| Less than 2 hours | Less than 6 hours | Less than 4 hours |
| --- | --- | --- |
|  |  |  |

1. What do you think the ‘Eatwell Plate’ shows us?

| How to eat lots of food | A healthy balanced diet | What not to eat |
| --- | --- | --- |
|  |  |  |

1. Which of these food groups do you think we should only eat occasionally?

| Bread , rice, potatoes, pasta and other starchy foods | Meat, fish, eggs, beans and other non-dairy sources of protein | Foods and drinks high in fat and/or sugar |
| --- | --- | --- |
|  |  |  |


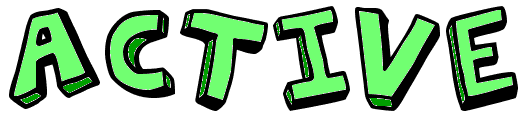

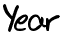

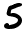

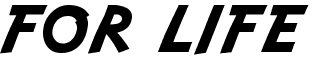


1. If you were trying to reduce the amount of TV you watched, what would be a healthy alternative?

| Play on a computer | Play outdoors | Surf the internet |
| --- | --- | --- |
|  |  |  |

1. Some snacks are healthy and can be eaten every day, other snacks should only be eaten occasionally (‘sometimes’ snacks). Which of these is an everyday snack?

| Chocolate bar | Crisps | Piece of fruit |
| --- | --- | --- |
|  |  |  |

1. How many portions of fruit or veg do you think you should aim to eat everyday to stay healthy?

| 1 | 5 | 3 |
| --- | --- | --- |
|  |  |  |

1. What is the main reason it is important to eat a healthy balanced breakfast to start the day?

| So you have lots of energy to last until lunchtime | To help you wake up | To keep your parents happy |
| --- | --- | --- |
|  |  |  |

1. Which of these ways of getting to school does **NOT** involve physical activity?

| Cycling | Walking | Travelling by car |
| --- | --- | --- |
|  |  |  |

**
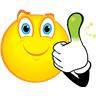
Thank you!**
